# Single-step dynamic dewatering of microalgae from dilute suspensions using flocculant assisted filtration

**DOI:** 10.1186/s12934-020-01472-4

**Published:** 2020-12-04

**Authors:** Mutah Musa, Andrew Ward, Godwin A. Ayoko, Christine Rösch, Richard Brown, Thomas J. Rainey

**Affiliations:** 1 Biofuel Engine Research Facility (BERF), School of Chemistry, Physics and Mechanical Engineering, Queensland University of Technology (QUT), 2 George Street, Brisbane, QLD 4000 Australia; 2 Innovation Centre, Queensland Urban Utilities (QUU), Main Beach Road Myrtletown, Pinkenba, Brisbane, QLD 4008 Australia; 3 Environmental Technologies Discipline, School of Chemistry, Physics and Mechanical Engineering, Science and Engineering Faculty, Queensland University of Technology (QUT), Brisbane, QLD 4000 Australia; 4 Institute for Technology Assessment and Systems Analysis (ITAS), Karlsruhe Institute of Technology, 76021 Karlsruhe, Germany; 5 Advanced Water Management Centre (AWMC), University of Queensland (UQ), St Lucia, Brisbane, QLD 4072 Australia

**Keywords:** Microalgae, Dewatering, Flocculants, Multi-criteria decision analysis, Chemometrics, PROMETHEE-GAIA

## Abstract

**Background:**

Dewatering constitutes a major challenge to the production of microalgae, accounting for 20–30% of the product cost. This presents a setback for the applicability of microalgae in the development of several sustainable products. This study presents an investigation into the dynamic dewatering of microalgae in a combined flocculation-filtration process. The effect of process conditions on the performance of 12 flocculants and their mixtures was assessed.

**Results:**

The mechanism of flocculation via the electrostatic path was dominated by charge neutralization and subsequently followed bridging in a ‘sweep flocculation’ process. Cationic polyacrylamide (CPAM) based flocculants recorded the highest biomass retention with PAM1 and PAM2 attaining 99 and 98% retention with flocculant dosages of 10 and 15 mg/L respectively. Polyvinylamine (PVAM) was also found to improve system stability across the pH range 4–10. Alum was observed to be only effective in charge neutralization, bringing the system close to its isoelectric point (IEP). Chemometric analysis using the multi-criteria decision methods, PROMETHEE and GAIA, was applied to provide a sequential performance ranking based on the net outranking flow (ф) from 207 observations. A graphical exploration of the flocculant performance pattern, grouping the observations into clusters in relation to the decision axis ($\pi$), which indicated the weighted resultant of most favorable performance for all criteria was explored.

**Conclusion:**

CPAM based flocculants and their mixtures demonstrated superior performance due to their viscoelastic behaviour under turbulence. The use of PVAM or alum in mixtures with CPAM reduced the required doses of both flocculants, which will provide beneficial financial impact for largescale microalgae dewatering in a flocculant assisted dynamic filtration process. Chemometric analysis based on the physico-chemical properties of the system provides a time saving assessment of performance across several criteria. The study findings provide an important foundation for flocculant assisted dynamic filtration processes.

## Background

Microalgae are a diverse group of unicellular organisms found in both marine and freshwater habitats, with inherent characteristics of a rapid growth rate. Microalgae have found relevant applications in wastewater treatment for organic pollutants degradation to produce clean water [1]; food and feed production [2]; CO_2_ sequestration [3]; and chemical feedstock for industrial products (e.g. biofertilizer and bioplastics [4, 5]). Furthermore, the high photosynthetic productivity of microalgae and its ability to be cultured on non-arable land or in the oceans (seawater), makes it attractive for commercial exploitation in meeting food, energy and climate security demands [6, 7]. Compared with higher (terrestrial) plants, microalgae have a greater ability to fix CO_2_ and produce biomass, which can potentially be used in making a wide range of products [6]. However, the industrial use of microalgae has been limited to high value products (e.g. pharmaceuticals), due to processing challenges at various stages. The significant challenge of economically dewatering microalgae is of particular concern to both academia and industry.

The common approach to dewatering microalgae is through a two-step process from dilute suspensions: (i) concentration of dilute suspensions to a slurry; and (ii) further dewatering the slurry to obtain a ‘cake’. These steps are referred to as primary and secondary dewatering respectively [8, 9]. The primary step aims at concentrating a suspension to a slurry of 2–7% biomass concentration, while the secondary further dewatering step could attain 15–30%, from which any further concentration will require an additional drying step [9].

Dewatering of microalgae from dilute culture suspensions at harvest is challenged by several drawbacks across the wide range of available technology currently being deployed. This challenge is further complicated by the small cell size that translates to a low specific gravity, and a negative surface charge that enables stability in colloidal suspension. Consequently, low biomass recovery efficiency, high capital costs and/or high operating costs have become common attributes of currently deployed dewatering techniques. For a myriad of reasons, it is difficult to apply conventional dewatering techniques for microalgae biomass [8].

The most commonly deployed dewatering technologies are briefly outlined. Sedimentation by inexpensive gravity settling is only suitable for microalgal species with a cell size > 70 µm and requires long retention time (1–2 days) [9]. The terms flocculation and coagulation have often been used interchangeably, with the main difference being that coagulation is irreversible whereas flocculation can be reversed [10]. Flocculation using flocculants improves sedimentation by inducing cell aggregation. In contrast, flotation involves the transport of microalgae cells to the surface of the culture solution using air bubbles and surfactants, which allows the biomass to be skimmed for surface collection [11]. Although flocculation and flotation reduce energy demand, they often achieve concentrations < 10%, and require the use of chemical additives (i.e. flocculants and surfactants) [12]. Non-synthetic sources of flocculants have also been explored and several bioflocculants are being applied for microalgae dewatering. Cationic starch and tanfloc, flocculants obtained from starch and tannin respectively are effective biopolymers in the flocculation of microalgae [13, 14]. Eggshell, known for its high CaCO_3_ content has also been applied in the flocculation of microalgae *C. vulgaris* with up to 99% efficiency [15]. Eggshell has been reported to compose of 94% CaCO_3_, in addition it has a cationic charge density, which makes it suitable for the destabilization of negatively charged particles like microalgae cells [16]. For these reasons, eggshell was explored as a bioflocculant in this study.

To achieve the desired higher biomass concentration (20–30%), centrifugation and filtration processes are often applied as secondary steps. However, centrifugation has a high capital expenditure and it is an energy intensive process that applies high centrifugal force that may adversely affect cell integrity [17]. Membrane micro- and ultra-filtration processes also attain high biomass concentrations, but are highly affected by membrane fouling and high cake resistance, requiring frequent membrane backwashing [12]. Novel methods being investigated include the use of magnetic nanoparticles, forward osmosis and ultrasound [18–20]. However, these novel methods are yet to be scaled on pilot and commercial scales.

The aim of this study was to apply the fibre dewatering principles used in paper making, and evaluate whether microalgae could be dewatered in a similar manner—i.e. dewatering from dilute suspensions at high rates under turbulence. This involved the dynamic dewatering of microalgae on the Britt dynamic drainage jar (BDDJ), an instrument designed to simulate the turbulent conditions of a commercial paper machine on a laboratory scale [21]. The effect of the microalgae culture suspension, experimental conditions (e.g. stirring speed and flowrate) and flocculant properties (e.g. molecular weight and dosage) on the efficiency of the dewatering process under turbulence was assessed, and subsequently correlated using a Multi-Criteria Decision Analysis (MCDA) technique i.e. chemometrics.

## Results and discussion

### Flocculant characterization

The physico-chemical properties of the flocculants were assessed from stock solutions of known concentration used in the study. Analysis were conducted at the Chemistry and Physical Sciences Laboraotory and the Central Analytical Research Facility (CARF) of the Queensland University of Technology (QUT). Table 1 presents a summary of the results. Fourier transform infra-red (FTIR) spectra were obtained from the flocculants in their powder forms, except for PolyA and PolyV which were analysed in liquid forms. Throughout the study the term flocculants refers to both forms of chemical additives (flocculants and coagulants).

**Table 1:** Physico-chemical properties of the flocculant stock solutions used in the study

Flocculant	Supplied form	Concentration (mg L^−1^)	Density (kg m^−3^)	Surface tension (mN m^−1^)	^a^Molecular weight (10^6^ Da)	^a^Charge density (mol%)	pH	Zeta potential (mV)	Viscosity (mPa s)
AL	Powder	10,000	1027	45.30	–	–	3.33	− 0.52	1.08
BN	Powder	10,000	1139	59.11	–	–	9.49	− 39.70	4.74
ES	Powder	20,000	1012	55.10	–	–	7.23	− 3.35	0.93
LC	Powder	20,000	1017	53.62	–	–	6.61	− 1.00	0.95
PAM1	Powder	2000	1059	66.27	Very high	Medium-High	4.49	84.00	19.17
PAM2	Powder	2000	1010	71.06	4.9–7.4	30	3.52	48.50	9.98
PAM3	Powder	2000	1025	56.76	High	Low-Medium	4.22	60.00	13.27
PAM4	Powder	2000	1036	70.88	Medium-High	Low-Medium	5.45	− 89.00	17.48
PAM5	Powder	2000	1146	51.46	Low	Low	6.50	− 83.70	16.58
PolyA	Liquid	40,000	1020	45.29	–	High	4.34	41.20	5.07
PolyV	Liquid	40,000	1009	63.91	–	High	8.12	31.53	4.48
SS	Powder	8000	1007	71.92	–	–	6.90	− 14.80	1.34

^a^Supplier advice values

The densities of the flocculant solutions ranged between 1007 and 1146 kg m^−3^ (see Table 1), all with values higher than the density of water. The higher densities are attributed to the fast settling observed with agglomerated microalgae when the non-polymeric flocculants were applied. The separation of solids in a liquid medium takes place rapidly when the density of the particles is markedly different from that of the liquid medium. This makes it easier for the particles to either settle out or float on top of the liquid. Difficulty arises when the particle size allows it to remain in suspension in the liquid medium as is the case with microalgae cells. Separation in such cases can be induced using flocculants.

Surface or interfacial free energy is an indicator of intermolecular bond strength existing as a result of cohesion between molecules at the surface of a fluid. Crushing a solid into pieces or dissolving it in a solution disrupts its bonds and therefore increases free energy. All flocculants used in the study had surface tension below that of water (72.19 mN m^−1^). SS, PAM2 and PAM4 had the highest surface tension of 71.92, 71.06 and 70.88 mN m^−1^ respectively (see Table 1), with values close to that of water. Reduction in surface tension has been reported to improve dewatering [22]. The stability of aqueous stabilized colloids (i.e. colloidal particles in an aqueous suspension) can also be affected by the surface tension of the aqueous system.

The intrinsic pH of the prepared flocculant solutions was found to affect system pH during dewatering, especially when strongly acidic or basic flocculants were applied [21]. Surface charge is another key interaction factor, which needs to be considered in dewatering studies [23]. Zeta potential is a measure of the electric potential at the shear plane between charged particles and the diffusion region called the electrical double layer [24], which gives a measurement of the apparent surface charge. Zeta potential is a physical property exhibited by any particle in suspension. The zeta potential of the flocculant stock solutions were estimated and later compared to values obtained for each observation in the experiments. The combination of zeta potential and pH can be used to tune the formulation of suspensions, in order to attain the isoelectric point (IEP) at which charge neutralization can be maximized. Therefore, an understanding of the culture medium-cell and cell-cell boundary interaction is important to optimising a dewatering process.

The stirring process that induces shear in a dynamic dewatering system is also known to have an effect on flocculant viscosity and efficiency. The stirring process allows even distribution of the flocculant within the microalgae suspension. This makes it easy for binding sites in the microalgae cells to get attached and form stable flocs. The cumulative effects of the outlined physicochemical properties on the dewatering process will be elucidated in the discussion section.

**Fig. 1:**
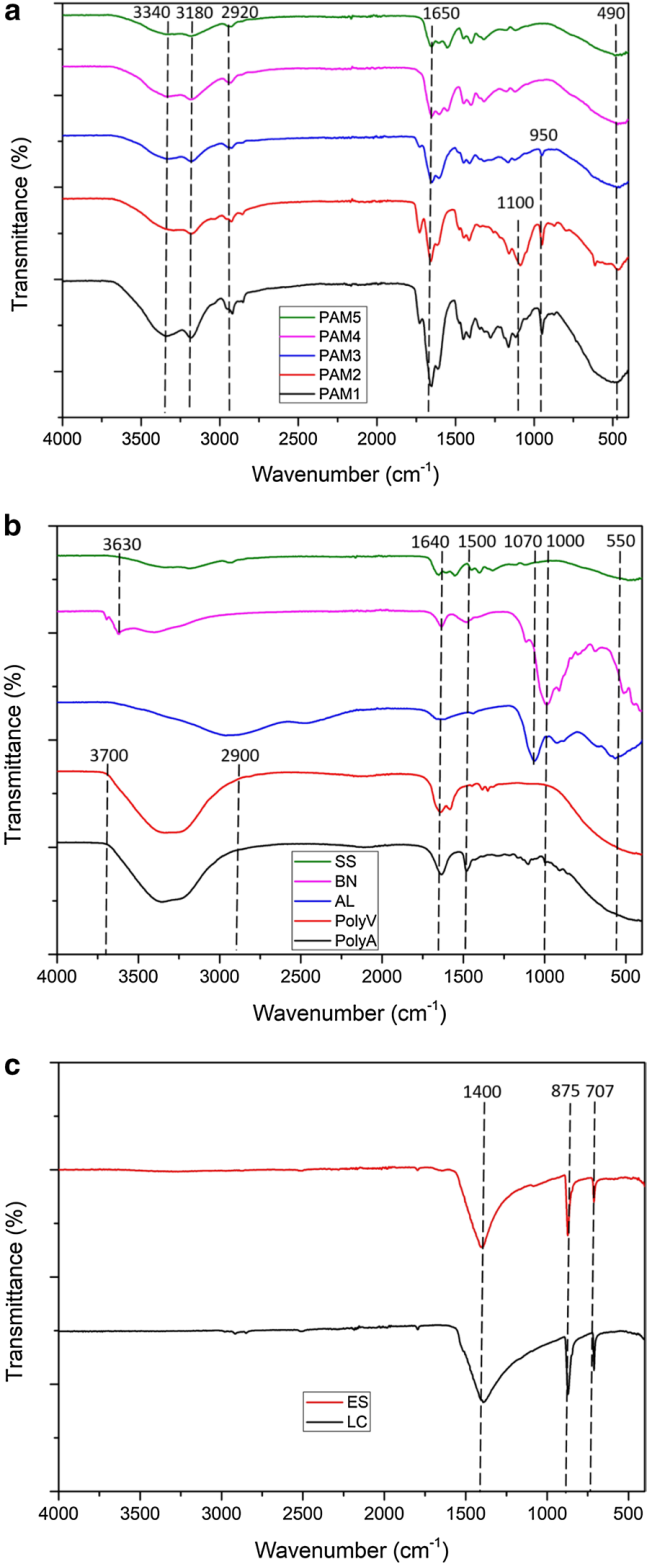
FTIR spectra of **a** polymeric flocculants, **b** non-polymeric flocculants and **c** comparison between eggshell and laboratory grade CaCO_3_

FTIR spectroscopy was performed within the mid-infrared region (4000–400 cm^−1^) to study the fundamental vibrations and associated rotational-vibrational structure of the molecules of the flocculants, and were grouped into three spectra graphs presented in Fig. 1. The main purpose of the assessment was to identify the active functional groups in each flocculant and subsequently to correlate the contribution of these groups to the dewatering performance, where these flocculants were applied. The FTIR technique basically characterises molecular structures, from which an assessment of chemical bonds and structural compositions of the flocculants could be made.

The peak trends were similar for all CPAM flocculants used in the study, with the major variation being the intensity of the peaks as presented in Fig. 1a. The broad stretching vibration peaks at 3340 and 3180 cm^−1^ were that of amide groups (–C=O –NH_2_) and –OH of carboxylic acids respectively [25, 26]. Hydrogen bonding effects, resulted in a decrease in the intensity of peaks across PAM1 to PAM5 [25]. Slight stretching vibrations observed at 2920 cm^−1^ were attributed to –CH of alkane groups, with vibration peaks of C=C also observed at 1650 cm^−1^. The peaks at 1450, 1400 and 950 cm^−1^ were that of –CH_2_N^+^(CH_3_)_3_ methylene groups [27]. However, the methylene group peaks at 950 cm^−1^ were only present in PAM1, PAM2 and PAM3 spectra. Furthermore, PAM1 and PAM2 had additional vibration peaks at 1160 and 1080 cm^−1^ corresponding to C–N stretches of amines. The peaks at 3340, 3180, 2920 and 1650 cm^−1^ are characteristic vibrations of the amide groups of CPAM [26]. The spectra provided sufficient information on the polymeric nature of the flocculants, and the observed increase in peak intensity as well as additional peaks corresponded with increase in both molecular weight and charge density.

In Fig. 1b PolyA and PolyV had broad wide peaks between 3700 and 2900 cm^−1^ attributed to N–H vibration stretches of amine groups and –OH of the solution [28]. The presence of amine groups at 1640 cm^−1^ and an additional methylene group were observed in the PolyV spectra [29]. For AL the bending mode of Al(H_2_O)_6_^3+^ was observed at 550 cm^−1^ and –OH bend at 1070 cm^−1^ [30, 31]. For BN the spectra had a sharp band at 3630 cm^−1^ due to the –OH stretching vibrations of structural OH groups, and the peak at 1000 cm^−1^ occupies similar position of OH bonds associated with structural hydroxyls [32]. For SS slight deformation vibrations at 3200 cm^−1^ and C–O–C stretching at 1660 cm^−1^ were observed [33].

In Fig. 1c the comparative spectra of the eggshell (ES) synthesized and laboratory grade CaCO_3_ (LC) are presented, with both having similar spectra patterns. Vibration peaks at 1400, 875 and 707 cm^−1^ corresponding to the in and out plane bending peaks and the asymmetrical stretching vibration peak of O–C–O carboxyl groups, which are associated with calcite [34].

### Effect of flocculant dosage on dewatering performance

**Fig. 2:**
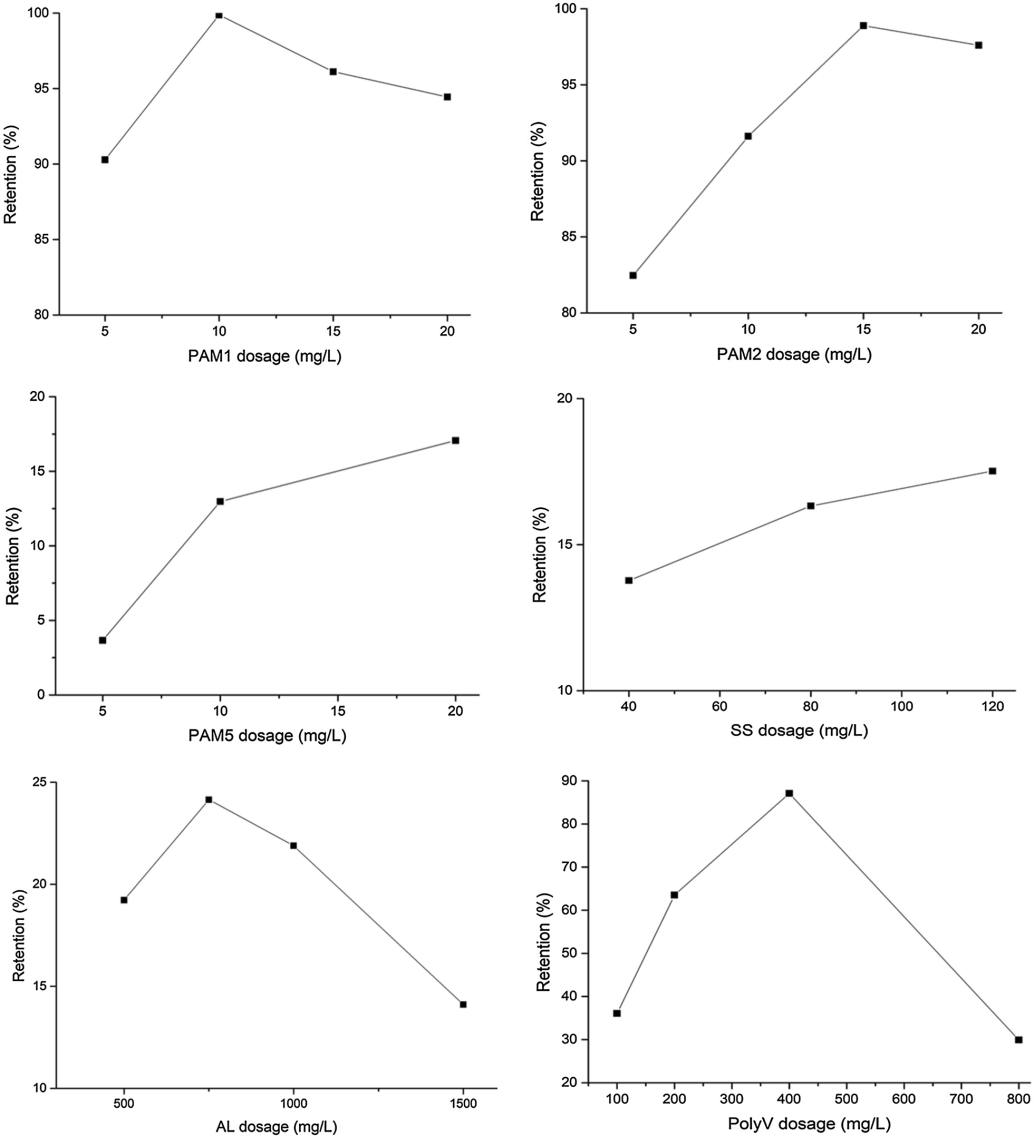
Effect of flocculant dosage on biomass retention

Figure 2 shows the effect of the addition of flocculants on microalgae dewatering performance on the BDDJ. Mixing the microalgae suspension with varying doses of the flocculants improved the efficiency of the process, although some flocculants surpassed others in terms of performance. There was an observed increase in retention with an initial increase in flocculant dosage, which peaks at varying concentrations depending on the flocculant type, until no further improvement in retention or a decline was observed. PAM1 attained a maximum retention of 99% at a dosage of 10 mg/L, while PAM2 achieved 98% at a dosage of 15 mg/L. For PolyV the maximum retention was 87% recorded at 400 mg/L. For PAM5, SS and AL included in Fig. 2, their dewatering performance was low. Other similar low performing flocculants were not included in Fig. 2. ES had retention rates of 17 and 11% at dosages of 200 and 300 mg/L respectively, with a similar performance observed with LC. PAM3 had retention rates of 29 and 43% at 10 and 20 mg/L respectively. The reported performance of PAM5, SS and AL could be attributed to their low molecular weights and charge densities. From the foregoing discussion, the behavior of the polymeric flocculants was observed to vary, with a possible explanation being the varying degrees of electroviscous effect. The electroviscous effect is the electrostatic contribution of particles to viscosity. This effect arises due to distortion of the electrical double layer around the particles during a shearing process and by changing the viscosity of the system via the introduction of polymeric flocculants [35].

The aggregation mechanism of low molecular weight flocculants occurs mainly via charge neutralization, and it required higher doses than that of medium to high molecular weight polymers [36, 37]. As microalgae cells have a negative surface charge that prevents aggregation, charge neutralization plays a vital role in reducing electrolytic repulsion. Electrolytic repulsion occurs via the dipole–dipole hydrogen bonding between the flocculant and the cells, and the interaction of van der Waals forces [38]. However, it was assumed that the repulsion forces were dissipated under turbulence. It has also been reported that increasing charge density is an effective means of controlling charge neutralisation [36].

In the case with high molecular weight flocculants, it is assumed that the interaction between the single cells of the microalgae in the cultivation solution and dosed flocculant was stronger. The results suggested that surface charge was highly increased when compared to the low molecular weight flocculants. This could be explained by the fact that the flocculants were high molecular weight and high charge density polymers, in which polymeric adsorption is upgraded by the charge differences in the solution system. Larger charge differences also implies rapid adsorption. High molecular weight polymers have also been ascribed as better bridging agents [39]. Another explanation could be drawn from the intensity of existing functional groups of the flocculants, which facilitated its adsorption on the microalgae cell surfaces, bridging the cells with resulting stable flocs [40]. The presence of amide groups (–C=O–NH_2_) in the lattice improves the process through the action of lone pair electrons from –N atoms of the amide binding unto more electronegative –O atoms [38]. As destabilization is achieved, cells with larger settling velocities will overtake smaller ones and the polymeric molecules of the flocculant attached to the surface of the cells links the cells together as they collide to form flocs, resulting in bridging [41]. The combined effects of charge neutralization and bridging induced by CPAM flocculants was found to significantly enhance turbulence resistance and consequently dewatering efficiency [41].

### Effect of pH and zeta potential on dewatering performance

**Fig. 3:**
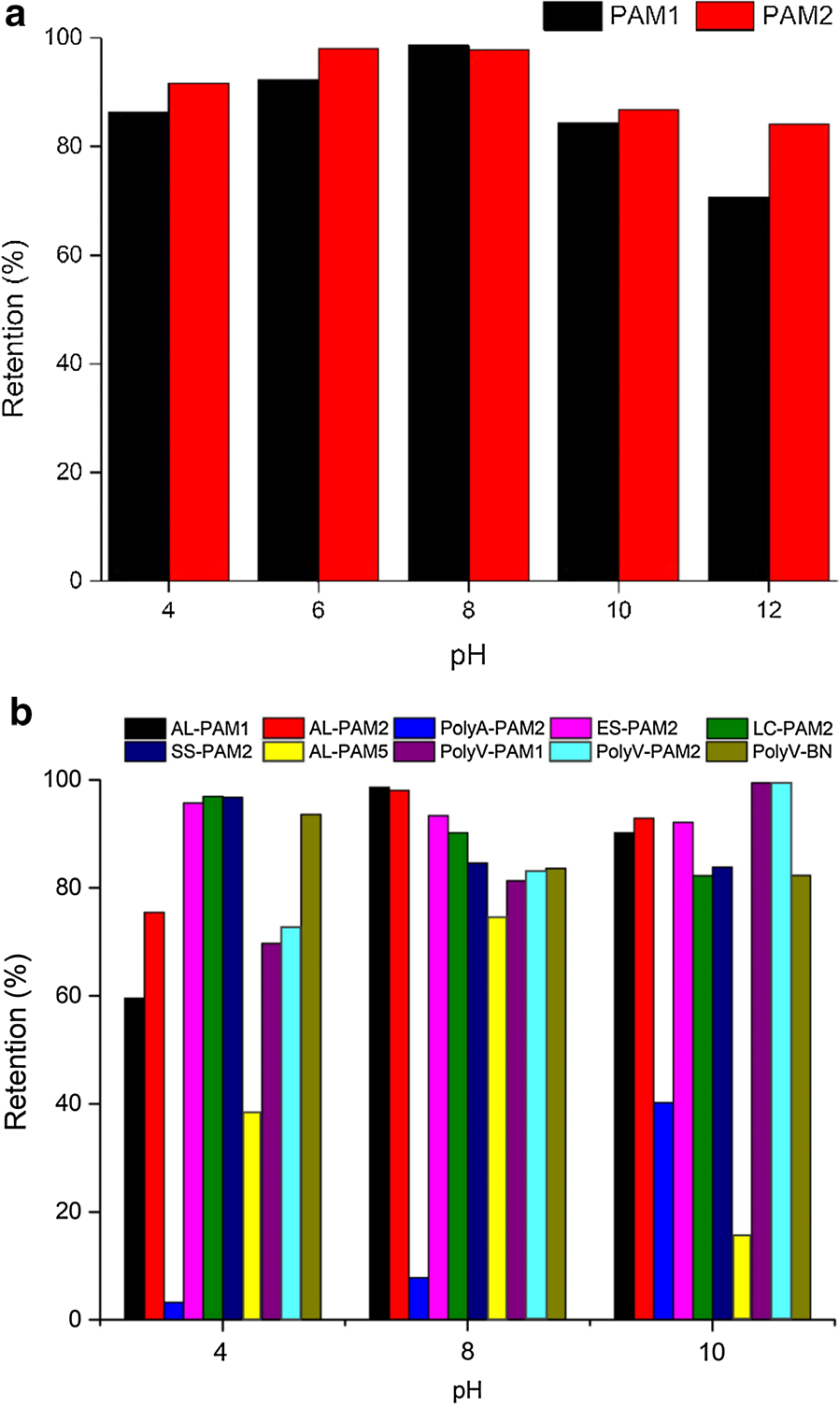
Effect pH on microalgae dewatering using **a** PAM1 and PAM2 and **b** double flocculant combinations

The system pH and zeta potential were studied in order to assess their contribution and effect on the efficiency of the dewatering process, as well as to regulate the dose of flocculants used in the study. Flocculant performance was analyzed at different pH values and the system zeta potential subsequently estimated. Biomass retention as a function of pH is presented in Fig. 3. Changes in pH were observed to significantly affect flocculation ability of the flocculants, consequently affecting dewatering performance. For the CPAM flocculants PAM1 and PAM2 that recorded high biomass retention, increase in pH initially improved their performance, and markedly declined beyond pH 8. Optimal retention (99 and 98%) were obtained at pH of 8 and 6 for PAM1 and PAM2 respectively. Floc formation was deterred at strongly acidic or alkaline states, from the performance of CPAM treated microalgae. However, PAM2 recorded retention rates > 80% in all pH ranges investigated in the study. Increasing the H^+^ ions enhanced positive charges in the system, resulting in increased electrostatic repulsion and colloidal stability [42]. While decrease in retention at high pH is associated to hydrolysis of the flocculant [43].

Subsequently, a process of combined flocculation was also investigated, where the performance of varying combination of flocculants was considered. Figure 3b shows the performance of various combinations at pH 4, 8 and 10. The trend between the mixes of PAM1 or PAM2 with other flocculants showed similar trend with that explained for PAM1 and PAM2 indicating that the flocculation process was dominated by the CPAM in the system. The combination is aimed at reducing the required total dose of both flocculants, making the dewatering process more economical [44]. Higher dewatering performance was observed with LC-PAM2 and SS-PAM2 at lower pH, this could be attributed to the additional stability contribution from Ca^2+^ and starch to the performance of the CPAM at low pH. The combination of AL and PAM5, which individually had low performance showed remarkable dewatering improvement. This implied that the charge neutralization by AL and bridging by PAM5 resulted in an improvement in dewatering. The precipitation of Mg^2+^ and Ca^2+^ ions at high pH has also been reported to effectively attract negatively charged microalgae cells [42]. Thus, the combination of ES and LC (with high calcium content) with PAM2 recorded remarkable retention rates (~ 80%). The combination of PolyV (a PVAM based flocculant) and PAM1 or PAM2 showed increase in performance with increase in pH from 4 to 10. The phenomena behind this observation in the behavior of PVAM was investigated by Nieto and co-workers [45]. The potential energy of polyamines are higher at pH 10, due the free space formed in the untangled polymer formation. On the other hand, higher charge density at pH 4 reduces interaction. This results in high protonation at pH 4 with a high content of crystalline zone, and as pH is increased crystallinity is reduced. At pH 10 optimum partial protonation is achieved, comprising both amorphous and crystalline zones with swelling of the polymer chains [45]. It can therefore be deduced that greater stability of CPAM-PVAM mix at pH 10 was due to the morphology blend between their partially amorphous and crystalline zones, which allows effective attachment of microalgae cells. However, in the case of the mixture of PolyV and BN, the net anionic charge of the bentonite particles of BN allowed more stability and higher dewatering performance at low pH. Therefore, it is important to state that the selection of flocculants to be used in a combined system should take into consideration the pH at harvest of the microalgae culture suspension. The *Dictyosphaerium* sp. used in this study had pH in the range 7.5–8.5 at harvest, and best dewatering performances were recorded between pH 6–8.

**Fig. 4:**
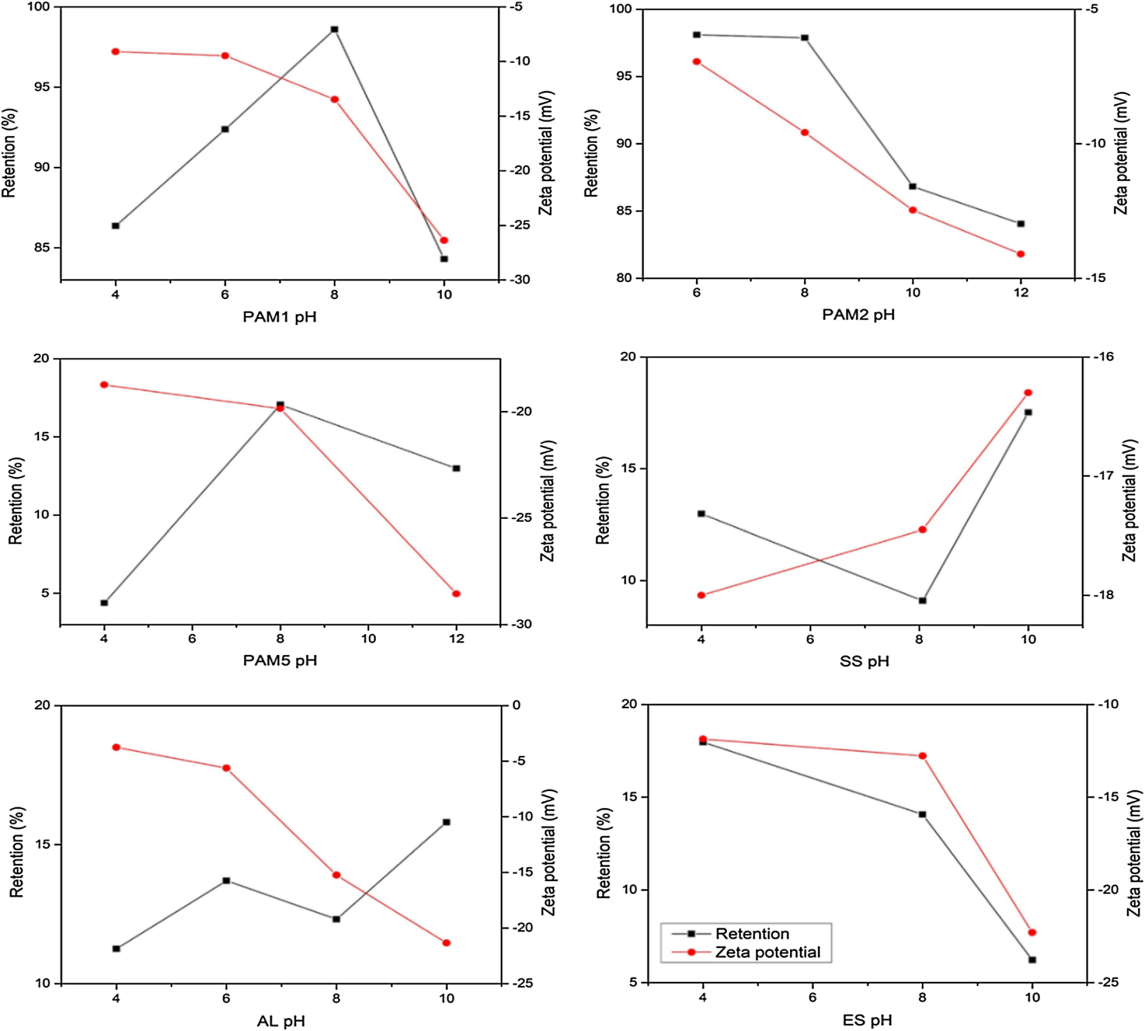
Effect of zeta potential on biomass retention with change in pH

Subsequently, the effect of zeta potential on dewatering performance was investigated. Figure 4 illustrates the effect of zeta potential on retention as pH changes. The zeta potential operational windows were observed to vary between − 4 and − 29 mV. Optimum retention using CPAM were observed when system zeta potential was above − 10 mV from the original culture suspension zeta potential of − 20 mV, this occurred at pH 8 and 6 for PAM1 and PAM2 respectively. In a previous study, it was found that zeta-potential values above − 10 mV were found to ensure optimum removal of natural organic matter using Ferric based flocculants, regardless of whether flocculant dosage or pH were altered [23]. In the case of AL where the highest zeta potential for all tested cases was recorded to be − 4 mV at a pH of 8, dewatering was below 20%. This can be explained by the high charge neutralization induced by the presence of aluminum hydroxide precipitates. In contrast, the high retention rates observed with CPAM at relatively lower zeta potentials (~ − 10 mv) suggests that dewatering performance is not only as a consequence of charge neutralization but also of the bridging, which occurs through sweep flocculation [23]. It has also been reported that while zeta potential could be useful in determining the flocculant dosage required to attain effective flocculation, there was a non-linear relationship between zeta potential and flocculation efficiency in microalgae species with complex morphologies (e.g. diatoms and colonies). Furthermore, it has also been reported that zeta potential measurements were not meaningful for polymer flocculated particles, as the effect of the polymer chains on the position of the effective ‘slipping plane’ is uncertain [23, 41]. The zeta potential at optimum dosage was lower than that attainable at IEP (i.e. zeta potential = 0 mV), indicating that effective dewatering was not entirely dependent on charge neutralization. If charge neutralization was the only flocculation path, then optimal efficiency will be achieved at or close to zero [22].

### Effect of stirring (turbulence) on dewatering performance

**Fig. 5:**
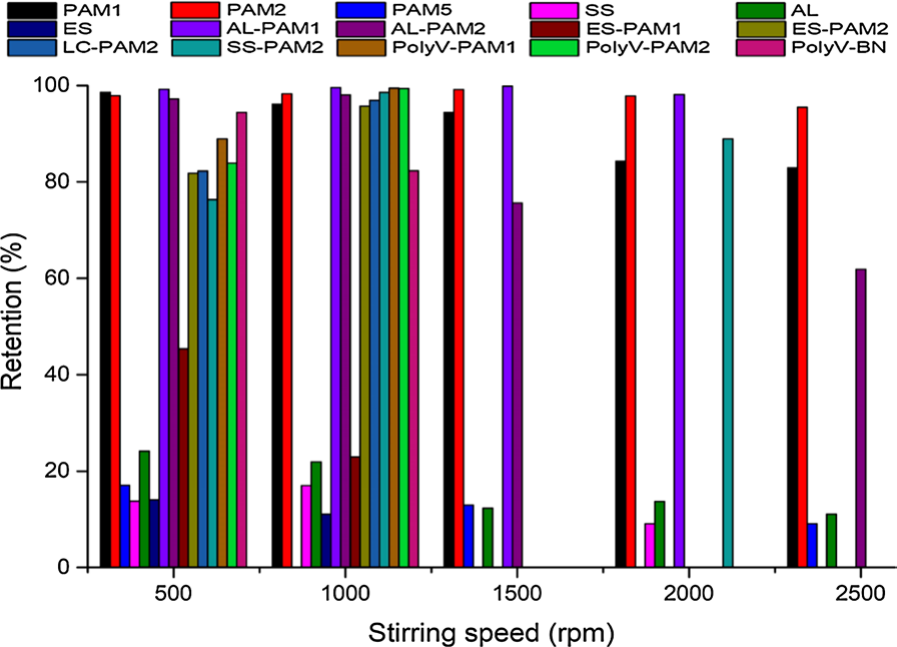
Effect of turbulence on biomass retention

The effect of stirring on the retention of microalgae is presented in Fig. 5. The turbulence induced by increasing stirring speed was observed to affect dewatering efficiency. From the preliminary experiments it was found that low stirring speed (< 500 rpm) did not produce sufficient mixing, thereby affecting the flocculant performance. Therefore, stirring speed in the range 500–2500 rpm was adopted for this study. The Technical Association of the Pulp and Paper Industry (TAPPI) test methods recommend stirring speeds between 500 and 1500 rpm for ideal headbox samples [46]. From the results, dewatering efficiency increased with an increase in stirring speed up to 1000 rpm, after which performance declined for most flocculants. As stirring speed was increased the flocculant molecules become open packed, creating an increase in void volume that allows microalgae cells to be adsorbed to the flocculant. However, the floc network collapses at higher speeds as the internal resistance of the flocculant reduces, permitting broken flocs to pass through the BDDJ screen. This behavior can also be explained by the interaction of present functional groups in polymers or their mixtures. The interaction between amide (–C=O–NH_2_) and the hydro-oxy (–OH) groups results in a congregation of molecules forming a structural (‘ball and sticks’) network with void pockets for microalgae cells to be attached or suspended in. As shear increases the hydrogen bonds are broken, collapsing the network and reducing viscosity [38]. A variant trend was however observed in the case the mixture AL-PAM1, which maintained high retention rates across all stirring speeds investigated in the study (500–2500 rpm). The could be attributed to the formation of high stability flocs with zeta potentials close to the IEP, combined with the viscoelasticity of CPAM.

### Floc morphology

**Fig. 6:**
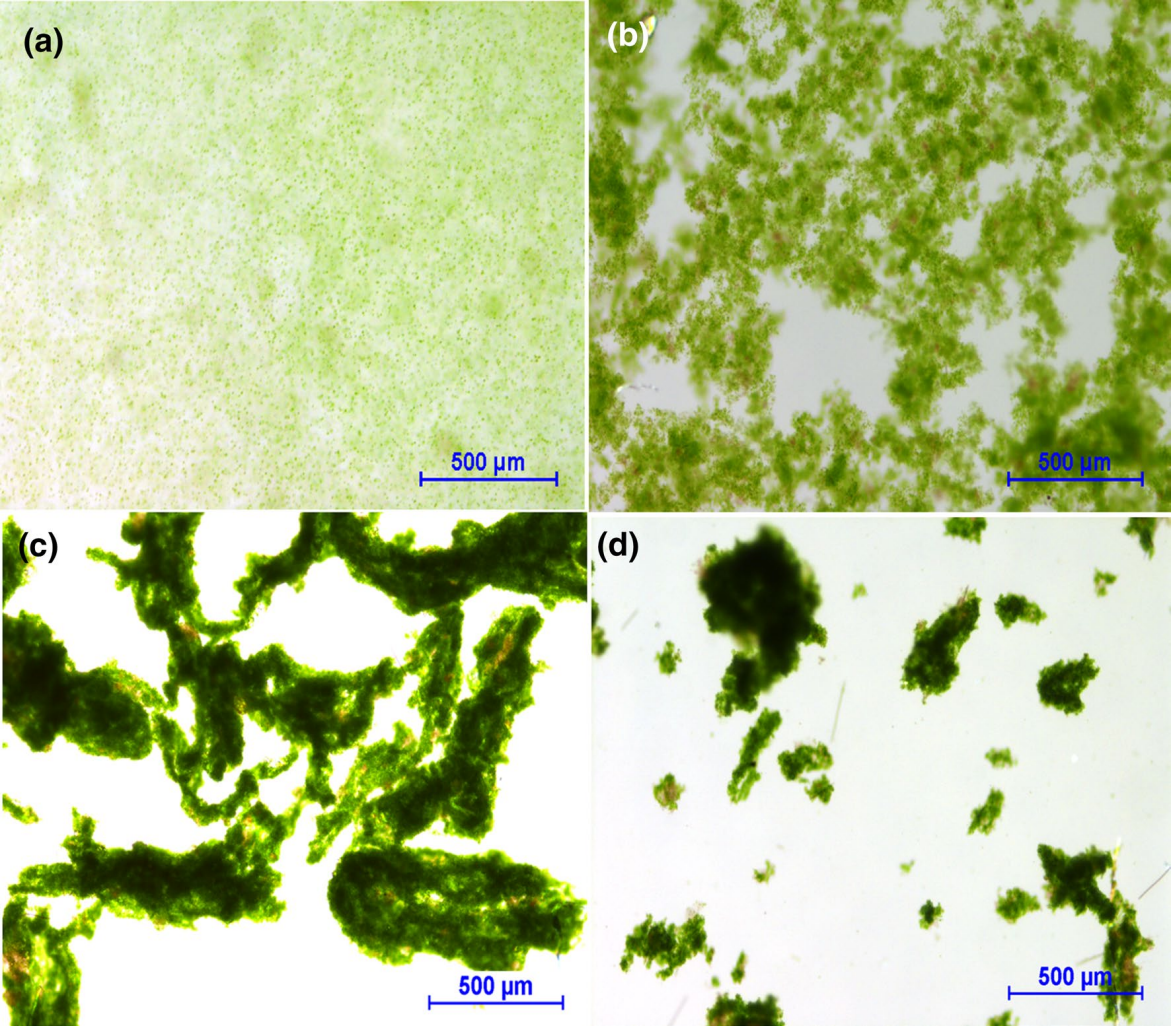
Micrographs of **a** unflocculated microalgae **b** alum flocculated microalgae **c** PAM1 flocculated microalgae and **d** PAM2 flocculated microalgae

Figure 6a presents the micrographs of unflocculated microalgae stirred at 1500 rpm and collected from the orifice of the BDDJ. While the micrographs in Fig. 6b–d were obtained under varying conditions with AL, PAM1 and PAM2 respectively. As a result of the variation in properties (e.g. chain length), different floc shapes and structures were developed with the flocculants applied in the study. Furthermore, shear-induced arrangement also plays a role in floc structuring. The micrographs show larger or longer floc forms with polymeric flocculants, which demonstrated higher stability under increased shear.

### Chemometric analysis

The foregoing discussions have elucidated the complexity of the interrelated effects of the various study criteria on dewatering performance, with the potentials for correlated synergistic or antagonistic contributions. Therefore, it becomes a cumbersome task to select one flocculant over the other because of the varying advantages across criteria. An MCDA approach using chemometrics makes the ranking of systems' performance possible, using Preference Ranking Organization METHod for Enrichment Evaluation (PROMETHEE) and Graphical Analysis for Interactive Aid (GAIA).

A scenario was designed to facilitate the ranking of the observations from the best to the worst using DecisionLab 2000. It is however important to provide DecisionLab with sufficient information related to the preferences of the decision maker (DM). This entails specifying the criteria as ‘maximized’ i.e. implying that observations with higher values were better performing, or ‘minimized’ i.e. implying that lower values were better performers. The preference functions for each criterion were selected from the six available options in DecisionLab. Table 2 presents the investigated criteria along with their preference settings. The ‘usual’ preference function in which no thresholds were considered and the linear (V-shaped) preference function were applied to the investigated study criteria (see Table 2). Each function could depend on up to two thresholds. The preference threshold P represents the smallest deviation between two observations that is considered decisive; Q the largest deviation that is considered negligible; and S is a value between P and Q used only with the Gaussian function. To enable a performance assessment on an incremented or decremented order, the criteria pH and zeta potential were scaled qualitatively, with scale ordering included in the supplementary material. The weight for each criterion was set to a value of 1, indicating that all criteria were considered to have equal importance.

**Table 2:** Dewatering assessment criteria, preference functions and weights for ranking by PROMETHEE

Criteria	FM^a^	pH	CFD^b^	FD^c^	SS^d^	BC^e^	R^f^	Q^g^	ZP^h^	OD^i^	MW^j^	BFR^k^	FCl
Unit			mg/L	mg/L	rpm		%	mL/s	mV	mm		%	AU$
Function Type^m^	Usual	Usual	Usual	Usual	Usual	Usual	Linear	Usual	Usual	Usual	Usual	Usual	Usual
Maximized	FALSE	FALSE	FALSE	FALSE		FALSE		FALSE	FALSE	FALSE	FALSE	FALSE	FALSE
Minimized	TRUE	TRUE	TRUE	TRUE	FALSE	TRUE	FALSE	TRUE	TRUE	TRUE	TRUE	TRUE	TRUE
P	2	2	2	2	2	2	10	2	2	2	2	2	2
Q	1	1	1	1	1	1	5	1	1	1	1	1	1
S	1	1	1	1	1	1	1	1	1	1	1	1	1
Scale	(Numerical)	Very good (1) Good (2) Poor (3) Very poor (4)	(Numerical)	(Numerical)	(Numerical)	(Numerical)	(Numerical)	(Numerical)	Very good (1) Good-Very good (2) Good (3) Poor-Good (4) Poor (5) Very poor (6)	(Numerical)	Very high (1) High (2) Medium-high (3) Medium (4) Low-medium (5) Low (6) Very low (7) None (8)	(Numerical)	(Numerical)
Weight	1	1	1	1	1	1	1	1	1	1	1	1	1

^a^FM: flocculant mix (where there were variations between one or two flocculant mixtures)

^b^CFD: combined flocculant dosage (i.e. total dosage of FM)

^c^FD: flocculant dosage for single flocculant system

^d^SS: stirring speed

^e^BC: biomass concentration in microalgae culture

^f^R: retention

^g^Q: filtrate flowrate

^h^ZP: zeta potential

^i^OD: orifice diameter

^j^MW: molecular weight

^k^BFR: biomass to flocculant ratio

^l^FC$: flocculant cost

The entire data set (207 observations i.e. experimental runs and 9 criteria) were analysed by PROMETHEE and GAIA, with the full results table included in the supplementary information (Additional file 1: Table S4). The higher the net outranking flow (ф) value for an observation, the higher its ranking and vice-versa. The model expectation therefore was that the PROMETHEE net outflow ranking would show a dominant trend of the best performing dewatering conditions, supported by flocculant performance. The relative range of PROMETHEE II net ranking outflow order was − 0.73 ≤ ф ≤ 0.72. The net outranking flow showed the spread of the observations, in such a way that the further apart the outranking flows were, the larger the degree of preference [47]. The relative scaling process facilitates a sensitive comparison between observations, rather than forcing an observation to be assigned a particular rank as found with conventional nominal rating scales [48]. Therefore, taking into consideration the scale of the thresholds and the precision of measurements (within experimental errors) it was possible to obtain a performance based ranking.

**Fig. 7:**
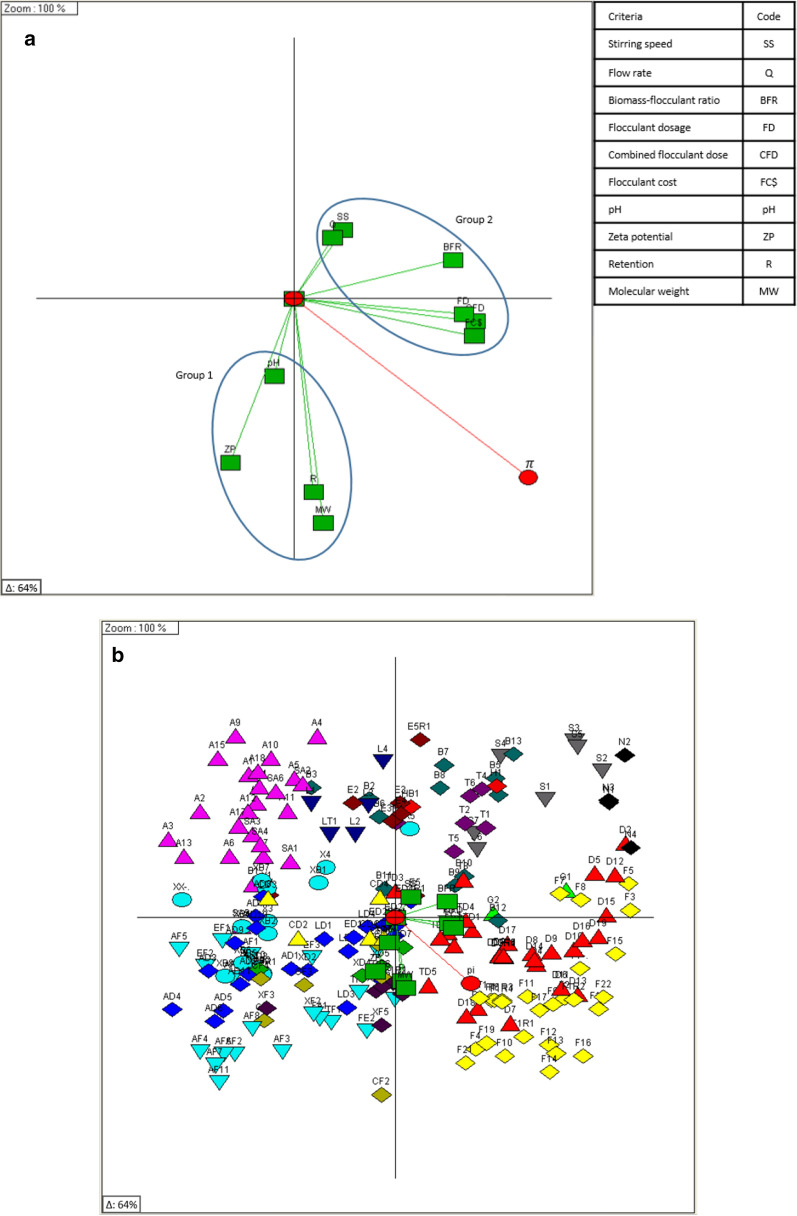

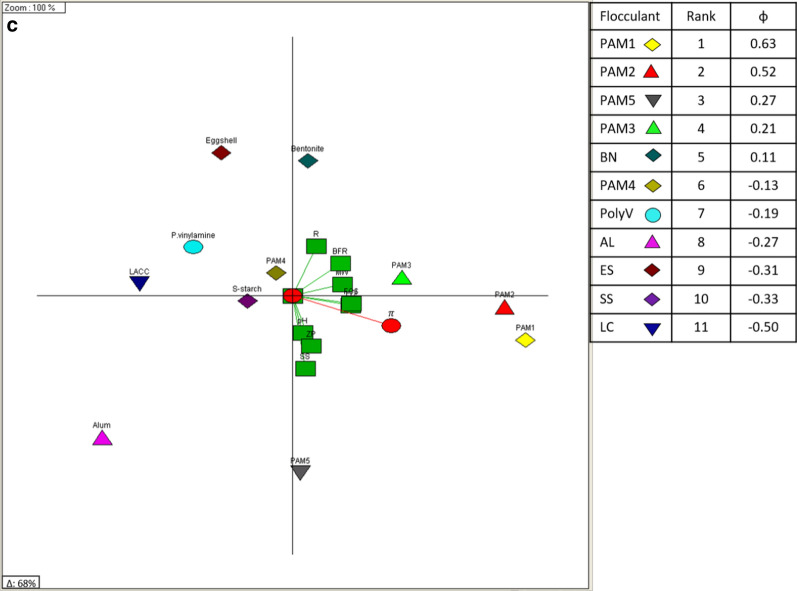
GAIA biplots for **a** criteria and decision axis ($\pi$), **b** full experimental observations and **c** single flocculants ranking

The GAIA biplot was used in further exploration of the pattern recognition. The GAIA biplot for the entire observations (207) across 9 criteria (Fig. 7) accounted for 64% of the data variance. The criteria vectors all roughly point in the direction of the $\pi$ decision axis in Fig. 7a (i.e. within an angle of 90°), with the length of the decision axis also indicating the robustness of the decision [48]. Criteria effects could be clearly separated into two groups, with Group 1 relating to chemical criteria and their resultant effects, and Group 2 relating to physical criteria including cost. The relationship between observations and the criteria further distinctly separated the observations by flocculant performance.

Low dewatering performance was recorded with AL [pink triangle], as indicated by observations located in an opposite direction to the $\pi$ decision axis (Fig. 7b). This could be explained by the fast charge neutralization where microalgae cell aggregates were only bound by weak electrostatic forces resulting from particle (cell) collision. The weakly bound aggregates were easily destabilized under turbulence, permitting passage through the 76 µm screen of the BDDJ (i.e. resulting in lower retention rates). Low performance flocculants were required at higher doses to yield this level of performance, often resulting in unsatisfactory retention rates (< 70%).

PAM1 [red triangle] and PAM2 [yellow diamond] the two leading flocculants, had observations distributed around the $\pi$ decision vector in Fig. 7b. The location of an observation in the $\pi$ direction indicates the quality of its performance in meeting the DM’s requirements. It was also observed that there was medium to high level of performance (80–95%) when varying the dose combinations of the other flocculants were used with CPAM (PAM1 or PAM2), taking advantage of their individual physicochemical properties and lowering process requirements, especially cost.

It was also evident that the observations formed clusters reflecting differences in flocculant type and performance (Fig. 7b). As the clusters were formed by flocculant types or their mixtures, it was possible to have preferential order of the clusters. Each cluster contained the strength and weakness of each flocculant across the investigated criteria. This provides a better understanding of the preferential structure of the partition to the DM, i.e. why one cluster could be preferred over another [49]. The preferential information contained in each cluster allowed the absolute ranking of the flocculants, reflecting their preferential quality as desired by the DM. Figure 7c presents an overall ranking of the flocculants investigated in this study, according to their dewatering performance by excluding mixtures, thereby improving the model’s efficiency in describing the data variability. From the ranking shown in Fig. 7c, it was derived that CPAM based flocculants of high molecular weight were able to form shear stable flocs that could withstand system turbulence and yield high retention at moderate to high turbulence levels.

**Fig. 8:**
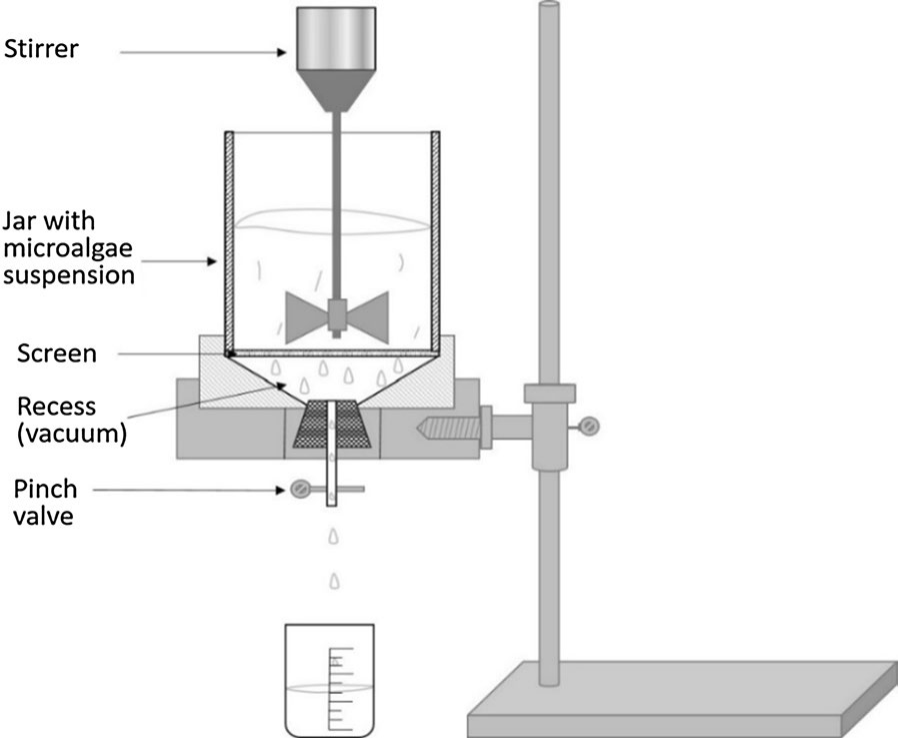
Britt dynamic drainage jar

Designing the dewatering process of microalgae suspensions and selecting the best performing flocculants is a complex task as several criteria have to be considered. The complexity is due to differing perspectives, values and preferences of decision-makers. To address this systematically, PROMETHEE-GAIA was applied as an example of a mathematical approach to measuring the overall effect of multiple criteria on the performance of the alternative flocculants. MCDA modeling was achieved in this study, by measuring the desirability of attaining different levels of performance in each criterion and combining these preferences across individual criteria allowing for inter-criterion comparisons. The PROMETHEE-GAIA results showed that, with some limitations, the mathematical approach is capable to assess multiple criteria holistically through pair wise comparison, to rank the investigated flocculants and to identify the most promising options among the flocculants. Beyond that, it allows for a graphic representation of the criteria using GAIA, which provides better understanding of the inter-dimensional interactions and conflicts of criteria, thereby facilitating consensus building in decision-making processes. The application of the PROMETHEE-GAIA methodology requires the application of certain functions. In particular, setting preferences for the selected criteria, since the results are influenced by the weights allocated to the criteria. The MCDA results can change if the evaluation process is performed using a default approach with the same weight for all flocculants or a tailored approach with different weights applied to assess performance of flocculants under specific conditions. To be applicable for general decision-making, the selected criteria should satisfy certain characteristics such as relevance, completeness, non-redundancy, understandability and feasibility. The criteria should also be clearly defined, judgmentally independent and scalable (i.e. measurable in an objective manner). As such, the relative importance of different criteria can be modelled by changing their weights or excluding or including criteria. The chemometric analysis provided a reliable outranking flow, based on the combined properties and performance of the flocculants investigated.

## Conclusions

The dewatering of microalgae was successfully explored on the BDDJ, an instrument which simulates a commercial scale paper machine. The results implied best dewatering performance for microalgae was achieved using CPAM. The flocculation mechanism path occurred by initial charge neutralization, followed by adsorptive bridging through sweep flocculation under electrostatic forces. The viscoelastic properties of CPAM contributed in maintaining the integrity of the flocs under turbulence. Modest to poor performance was recorded with low molecular weight CPAM and other non-polymeric investigated flocculants, mainly due to the weak binding of the microalgae cells, resulting in low turbulence tolerance.

Mixing of non-polymeric flocculants with CPAM was found to improve dewatering performance. The ultimate aim of mixing was for the low-cost flocculants to reduce the quantity of the high cost flocculants required for effective dewatering, while taking advantage of the inherent properties of both. This combined effect and performance are expected to bestow the dual benefits of environmental safety and economic profit, especially for commercial scale dewatering.

A sequential chemometric analysis was conducted using PROMETHEE and GAIA to identify the overall best alternative among the range of flocculants applied for microalgae dewatering. To the authors’ knowledge, this is the first time an attempt has been made to assess and compare these flocculants using PROMETHEE and GAIA. The results demonstrated that dewatering performance could be ranked from the system properties independent of conventional rating scales. Presentation of the information as a biplot, identified performance patterns that were related to conventional trends observed. The observations also formed clusters based on dewatering performance, which allowed a ranking of the flocculants. The use of additional criteria, such as environmental impacts or weight allocation to the considered criteria are other changes that can influence the ranking process.

This study does not only offer valuable insight on the performance of the various flocculants, but also provides an important foundation for flocculant assisted dynamic filtration processes. The use of a flocculant or combination of flocculants in a system tailored on the knowledge of the properties of both the microalgae cells (e.g. cell morphology) and the culture solution (e.g. pH at harvest) will be key to safe and economic microalgae dewatering.

## Materials and methods

### Analytical instruments

BDDJ equipped with 1 L jar supplied by Paper Research Materials Inc. (WA, USA); Aqua-pH meter supplied by TPS (QLD, Australia); Glass fiber filter paper (GA-55) from Advantec (CA, USA); Cary 60 UV–Vis spectrophotometer manufactured by Agilent Technologies (CA, USA); GR-200 analytical balance manufactured by AND (CA, USA); OM550 oven manufactured by Clayson Laboratory Apparatus Pty Ltd (QLD, Australia); Light microscope M125 supplied by Leica Microsystems Pty Ltd. (NSW, Australia); FTIR spectrometer Alpha-P manufactured by Bruker Scientific (CA, USA); MCR302 rheometer manufactured by Anton Paar (Graz, Austria); and Sigma 702 tensiometer KSV Instruments (Helsinki, Finland).

### Microalgae and culture conditions

A freshwater microalgae culture of *Dictyosphaerium* sp. was cultivated under phototrophic nutrient replete conditions in a 4.5 m^2^ surface area high rate algae pond (HRAP). The HRAP had a 800 L working volume and a 185 mm working depth. The culture was continuously mixed by a paddlewheel to attain the optimum culture mixing speed of 0.2 m s^−1^. The HRAP was located outdoors at the Queensland Urban Utilities (QUU) water treatment facility at Pinkenba, Brisbane, Australia. The culture was grown on high strength digestate produced from a waste activated sludge anaerobic digestion process. Wastewater has been identified as suitable medium for microalgae production and the use of wastewater as a nutrient source is seen as an advantageous medium to produce algal biomass, due to its abundant nutrients. The digestate wastewater feed had the following average chemical composition; K: 231 ± 77, Mg: 23 ± 3, Na: 437 ± 100, P: 162 ± 67, S: 21 ± 4 Ca: 35 ± 6, NH_4_ 847 ± 391 and SCOD: 369 ± 183 mg L^−1^. Due to the high ammonia strength associated with the digestate, digestate was diluted with potable water to give a final effluent concentration of approximately 500 mg L^−1^ ammonia –N. Microalgae was grown under semi batch conditions and was harvested when the cell count had reached its maximium cell count of 50–60 cells × 10^6^. Microalgae 10–15% V/V was left in the raceway during the harvesting process, this residual volume acted as the inoculm for the next semi batch growth period. The harvested microalgae biomass culture was stored at room temperature after harvest prior to processing. The microalgae biomass culture used in the study was harvested in five batches with concentrations between 233–301 mg/L. The experiments were performed directly without any form of pre-processing of the culture. For each sampling batch, all microalgae biomass culture collected were analyzed within 48 h of harvest.

### Flocculants

The terms flocculation and coagulation have often been used interchangeably, with the main difference being that coagulation is irreversible whereas flocculation can be reversed [10]. Throughout the study the term flocculants refers to both forms of chemical additives. All flocculants used in the study were obtained from commercial suppliers and stock solutions prepared at varying concentrations, with the exception of eggshells which were deproteinised for use at the Chemistry and Physical Sciences Laboratory at QUT. The flocculants used in single and combined states included cationic polyacrylamide (CPAM; a common flocculant for paper making), alum, laboratory grade soluble starch (Ajax Chemicals, Australia: CAS No. 9005-25-8), bentonite particles, laboratory grade calcium carbonate (CaCO_3_) (Ajax Chemicals, Australia: CAS No. 471-34-1), polyamine and polyvinylamine (PVAM) solutions. Code names were assigned to the flocculants to dissociate them from their commercial brand names, as presented in Table 3.

**Table 3:** Flocculants used in the study

Code name	Description
AL	KAl(SO_4_)_2_ (alum)
BN	Bentonite particle powder
ES	Deproteinized eggshell
LC	Laboratory grade CaCO_3_
PAM1	Cationic polyacrylamide based powder of high molecular weight
PAM2	Cationic polyacrylamide based powder of high molecular weight
PAM3	Cationic polyacrylamide based powder of high molecular weight
PAM4	Cationic polyacrylamide based powder of medium–high molecular weight
PAM5	Cationic polyacrylamide based powder of low molecular weight
PolyA	Polyamine based liquid flocculant (with 50% active content)
PolyV	Polyvinylamine based liquid flocculant (with 20% active content)
SS	Laboratory grade soluble starch powder

### Methods

The experimental study was conducted in three phases, viz.: (i) Flocculants characterization; (ii) Investigation of the effect of flocculants on the single-step dewatering of microalgae; and (iii) chemometric analysis.

### Eggshell preparation

Eggshell was prepared following similar methods used in previous studies [15, 50], and applied as a natural flocculant in this study. The eggshell was collected, washed and rinsed with UPW before oven drying at 45 ˚C for 76 h. The dry eggshell was ground down to a powder and mechanically sieved using a 325 µm mesh size. The eggshell was deproteinized by adding 100 g of the eggshell powder to a 1 L solution of 3.5% (w/v) NaOH and continuously stirred at 70 °C for 2 h. The sample was vacuum filtered and washed several times with UPW, before oven drying at 60 °C for 24 h. For comparison, laboratory grade CaCO_3_ was also applied for dewatering microalgae in known standard concentrations and its performance compared with that of the eggshell.

### Biomass concentration measurement

Biomass concentration was determined by suspended solids dry weight measurements. A 10 mL aliquot of the microalgae suspension was filtered through a glass fiber filter paper (GA-55 Advantec, USA), and rinsed several times with UPW. The filter was then placed in oven at 80 °C for 24 h.

### Flocculants characterization

The physico-chemical properties of the flocculants, which provided further details about their behavior and contribution towards the flocculation process were assessed. These properties included density; Fourier transform infrared (FTIR) spectra; solution surface tension; and viscosity measurements.

### Flocculant cost analysis

A cost analysis was conducted to quantify the cost of flocculating microalgae using the selected flocculants. Budgetary bulk price estimates were obtained from the manufacturers or bulk vendors of the industrial chemicals. For eggshells an estimate cost of collection from food processing industries and deproteinization was calculated and applied (prices current at May, 2019). All flocculant costs were calculated in Australian dollars (AUD), based on the flocculant dose required to dewater 50 m^3^ of microalgae suspension.

### Britt dynamic drainage jar dewatering experiments

The dewatering of microalgae culture suspension using the BDDJ was investigated in batch mode, following the Technical Association of Pulp and Paper Industry (TAPPI) method T261 [46], with modifications to accommodate microalgae. Briefly, 500 mL of homogenously mixed microalgae suspension was fractionated into the jar with the orifice closed. The BDDJ is schematically presented in Fig. 8. Details of the procedure can be found in an earlier study by the same authors [51].

Prior to the BDDJ experiment, jar settling tests were conducted to select the range of flocculant doses that was applied in the study. A 50 mL volume of microalgae suspension was fractionated into a measuring cylinder, and dosed with a known concentration of the flocculant. The mixture was allowed to settle (flocculate) over a period of 30 min. This was repeated several times for varied doses. After verifying the sufficient dosage for each flocculant, the BDDJ experiments commenced to determine the flocculants’ suitability under turbulence. Furthermore, where pH adjustment was required, 0.5 M solutions were prepared from KOH pellets (85% assay) and HCl (32%) for pH adjustment.

### Analytical methods

#### Biomass retention

The efficiency of the dewatering process was measured as the percentage of biomass retained on the 76 µm screen after each filtration run. This was determined via OD_750_ measurements of the filtrate to obtain retention rates. OD_750_ which is a measure of light absorbance was used, as it is a standard measure of biomass yield (i.e. retention in this study), and is highly correlated to residual biomass concentration [52]. The percentage retention was obtained from the relationship in Eq. (1).

1 $${\text{Retention}}\;{\text{(\% ) = }}\left( {1 - \frac{{\text{A}}}{{\text{B}}}} \right) \times 100$$

where *A* is the *OD*_*750*_ of the filtrate collected from the orifice of the BDDJ, and *B* is the initial *OD*_*750*_ of the microalgae suspension.

#### Zeta potential

The zeta potential of the flocculated microalgae systems were monitored during the dewatering process using Zetasizer Nano ZS series (Malvern, Australia). Microalgae samples were taken from the BDDJ in 10 mL vials, from which 1 mL uniformly suspended mix was injected into a folded capillary tube (DTS 1070) for zeta potential measurement. Measurements were performed in triplicate. The Zetasizer measures the electrophoretic mobility (EM), which is converted to zeta potential (ζ) using the Smoluchowski equation, given as Eq. (2).

2 $${\text{EM = }}\frac{{\varepsilon \zeta { }}}{{\mu }}$$

where ε and µ are the permittivity and viscosity of the solution respectively [23].

#### Microscopy

An optical microscope (Nikon Eclipse Ni, Japan) was used to observe the morphology of formed flocs. Flocculated microalgae specimens were prepared on a glass slide and viewed using varying magnifications to observe floc structure.

#### Chemometrics

All analytical data collected from instrument measurements were entered into Microsoft Excel spreadsheets and used as data inputs for the chemometric analysis. Information collected from 207 observations (dewatering experimental runs) included properties of: the flocculant (type, dosage, molecular weight and cost); instrument and system (stirrer speed, pH, zeta potential, flowrate and orifice size); and microalgae culture (pH and biomass concentration). The collected and organized data were subjected to MCDA by the Preference Ranking Organization METHod for Enrichment Evaluations (PROMETHEE) and Graphical Analysis for Interactive Aid (GAIA) procedures for multivariate data sets using ‘Decision Lab 2000′ software (DecisionLab). The mathematical algorithm and application procedures for PROMETHEE and GAIA have been described in the literature [48, 53, 54]. The procedures are based on non-parametric methods for the pairwise comparison of objects and variables.

## Supplementary information

**Additional file 1: Table S1.** Preference function types, definitions and shapes. **Figure S1.** Preference function *P(d)*. **Table S2.** Molecular weight scale. **Table S3.** pH scale. **Table S4.** Complete PROMETHEE rank order from the 207 observations.

## Data Availability

A significant part of the data generated and analysed in this study have been included in the article and its supporting information. Any further details about datasets used and analysed are available from the corresponding author on reasonable request.

## Abbreviations

AUD: Australian dollars
BDDJ: Britt dynamic drainage jar
CPAM: Cationic polyacrylamide
DM: Decision maker
EM: Electrophoretic mobility
ERT: Emission reduction target
GAIA: Graphical Analysis for Interactive Aid
GHG: Greenhouse gas
HRAP: High rate algae pond
IEP: Isoelectric potential
MCDA: Multi-criteria decision analysis
NOM: Natural organic matter
OD_750_: Optical density measured at a wavelength of 750 nm
OR: Operations research
PCA: Principal component analysis
PROMETHEE: Preference Ranking Organization Method for Enrichment Evaluation
PVAM: Polyvinylamine
rpm: revolutions per minute
TAPPI: Technical Association of the Pulp and Paper Industry
UPW: Ultrapure water
