# Supplementary material for: Single-step dynamic dewatering of microalgae from dilute suspensions using flocculant assisted filtration
Source: Microb Cell Fact. 2020 Dec 4;19:222. doi: 10.1186/s12934-020-01472-4 (PMC7716443; doi:10.1186/s12934-020-01472-4)
Supplement: Supplementary file 1 — Additional file 1: Table S1. Preference function types, definitions and shapes. Figure S1. Preference function P(d). Table S2. Molecular weight scale. Table S3. pH scale. Table S4. Complete PROMETHEE rank order from the 207 observations. [file 12934_2020_1472_MOESM1_ESM.docx]

**Single-step dynamic dewatering of microalgae from dilute suspensions using flocculant assisted filtration**

Mutah Musa^1,5^, Andrew Ward^2,5^, Godwin A. Ayoko^3^, Christine Rösch^4^, Richard Brown^1^, Thomas J. Rainey^1^*

^1^Biofuel Engine Research Facility (BERF), School of Chemistry, Physics and Mechanical Engineering, Queensland University of Technology (QUT), 2 George street, Brisbane, QLD 4000, Australia

^2^Queensland Urban Utilities (QUU), Innovation Centre, Main Beach Road Myrtletown, Pinkenba, Brisbane, QLD 4008, Australia

^3^Nanotechnology and Molecular Science Discipline, School of Chemistry, Physics and Mechanical Engineering, Science and Engineering Faculty, Queensland University of Technology (QUT), Brisbane, QLD, 4000, Australia

^4^Institute for Technology Assessment and Systems Analysis (ITAS), Karlsruhe Institute of Technology, 76021 Karlsruhe, Germany

^5^Advanced Water Management Centre (AWMC), University of Queensland (UQ), St Lucia, Brisbane, Queensland, 4072, Australia

*Corresponding author. Email address: [t.rainey@qut.edu.au](mailto:t.rainey@qut.edu.au) (T. J. Rainey)

Keywords: microalgae, dynamic dewatering, flocculants, Multi-Criteria Decision Analysis (MCDA), chemometrics, PROMETHEE-GAIA

**Supplementary Information**

1. **Multi-criteria Decision Analysis background**

Multi-criteria analysis originated as a sub-discipline of operations research (OR) that is applied in the evaluation of multiple criteria in decision making and it has found relevance in a wide range of areas (e.g. business and medicine) [1]. The extraction and correlation of physical and chemical information from a process, in order to understand their effect on the process or the outcome of the process is called chemometrics. In contrast to the wide scope of MCDA in operations research, chemometrics is considered to be a chemistry discipline. However, chemometric methods provide a backdrop on which the concepts and contributions of MCDA could be compared and understood [2]. Chemometrics is currently being applied in chemical, environmental, industrial and engineering process analysis [3].

1. **Preference functions**

The preference function translates the deviation between the evaluations of two observations on a single criterion, in terms of the degree of preference. Six specific shapes have been proposed to facilitate the association of a preference function to each criterion.

Table S1: Preference function types, definitions and shapes

| **Type** | **Shape** | **Definition** | **Parameters** |
| --- | --- | --- | --- |
| 1. Usual criterion | 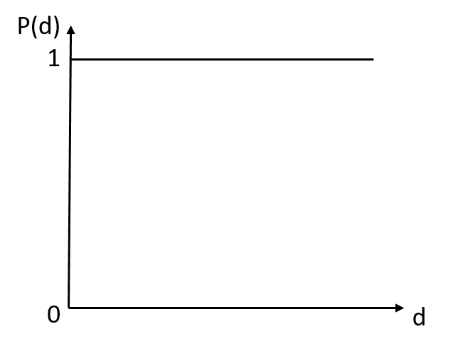 | $P\left( d \right)=\left\{ \begin{aligned} 0 \text{d}\leq0 \\ 1 \text{d}>0 \end{aligned} \right.$ | - |
| 1. Quasi-criterion | 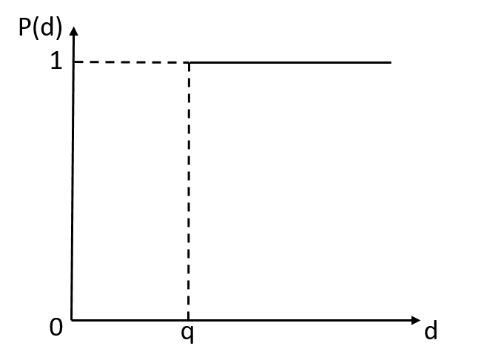 | $P\left( d \right)=\left\{ \begin{aligned} 0 \text{d}\leq q \\ 1 \text{d}>q \end{aligned} \right.$ | q |
| 1. Criterion with linear preference | 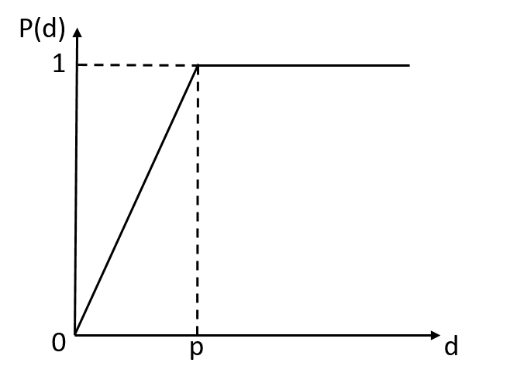 | $\text{P(d)=}\left\{ \begin{matrix} 0 \text{d}\leq0 \\ \frac{d}{p} \text{0}\leq d\leq p \\ 1 \text{d}>p \end{matrix} \right.$ | p |
| 1. Level criterion | 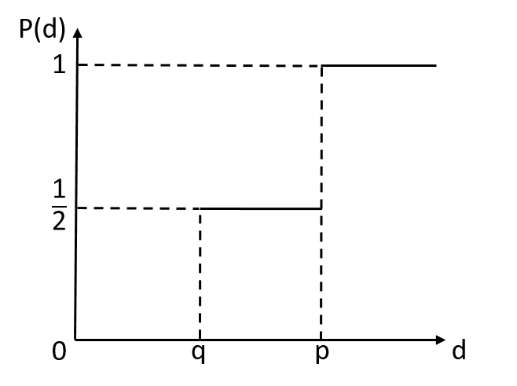 | $\text{P(d)=}\left\{ \begin{matrix} 0 \text{d}\leq0 \\ \frac{1}{2} \text{q}<d\leq p \\ 1 \text{d}>p \end{matrix} \right.$ | q, p |
| 1. Criterion with linear preference and indifference area | 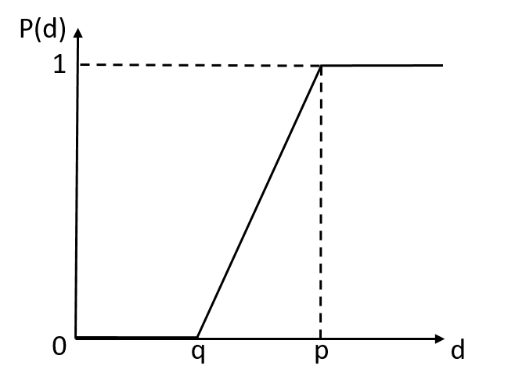 | $\text{P(d)=}\left\{ \begin{matrix} 0 \text{d}\leq q \\ \frac{d-q}{p-q} \text{q}<d\leq p \\ 1 \text{d}>p \end{matrix} \right.$ | q, p |
| 1. Gaussian criterion | 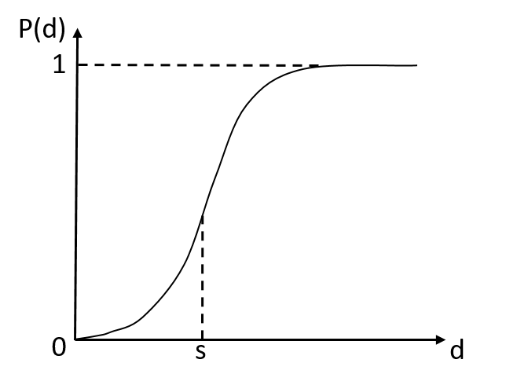 | $P\left( d \right)=\left\{ \begin{aligned} 0 \text{d}\leq0 \\ 1-e^{-\frac{d^{2}}{{2s}^{2}}} \text{d}>0 \end{aligned} \right.$ | s |

1. **PROMETHEE and GAIA procedures**

PROMETHEE facilitates the ranking or ordering of objects (in this study flocculants) according to preference and weighting conditions which have been selected and applied to the variables (e.g. cost, pH and molecular weight). The first step involved selection of the preference function, which provides the basis on which one object is preferred over another. In commercially produced software such as the one used in this study i.e. DecisionLab 2000, six preference functions are available in the procedures, details of which are included as supplementary information. These functions allow an assignment of threshold values for each variable used in the study. It is also necessary to indicate if higher or lower criteria values are preferred by nominating a rank order to either ‘maximize’ i.e. top-down or ‘minimize’ i.e. bottom-up. To further refine the selection process, positive (ф+) and negative (ф-) outranking flows are computed from the $\pi$ indices in PROMETHEE. The former expresses how each object (flocculant) outperforms all others, and the latter indicates how an object is outperformed by all others. Subsequently, a simple set of procedures (included in supplementary information) is applied to compare the outranking flows, and a partial ranking (PROMETHEE I) or a complete ranking (PROMETHEE II) is computed.

GAIA is a procedure that uses principal component analysis (PCA) techniques to evaluate and graphically display PROMETHEE results. GAIA reduces a large number of variables into two of principal components and shows visually how variables relate to each other and the objects [4]. This results from the mathematical decomposition of the net outranking flows ф, such that observations were regarded as actions and the variables in the data matrix as criteria. This allowed clustering of actions and identification of outliers from the GAIA biplots. Furthermore, GAIA has a spreadsheet format conformation that allows for sensitivity analysis, by weighting each criterion [1]. The advantages of GAIA over other multivariate analysis methods is that (i) it allows an interpretation of the performance of each object across all criteria using the $\pi$ decision axis that appears in the biplot as a weighted resultant of all the criterion axes; and (ii) it does not require a pretreatment (data reduction and standardisation) procedure, as PROMETHEE serves as a pretreatment for GAIA [5, 6].

PROMETHEE is a non-parametric method that ranks a number of observations across a range of criteria. The following considerations are applied in the ranking algorithm.

1. A preference for higher or lower criteria values is indicated by assigning for each variable a rank order to either ‘maximize’ or ‘minimize’ its values.
2. For each criterion considered in the model, all column entries in the data matrix (relating to observations) are compared pairwise by subtraction in all possible combinations, to obtain the difference (*d*) for each comparison.
3. The preference function (*P*) selected for each criterion is then used to allocate a preference value to each *d*. This is given by the general relationship in Figure S1, and the specific types already given in Table S1.


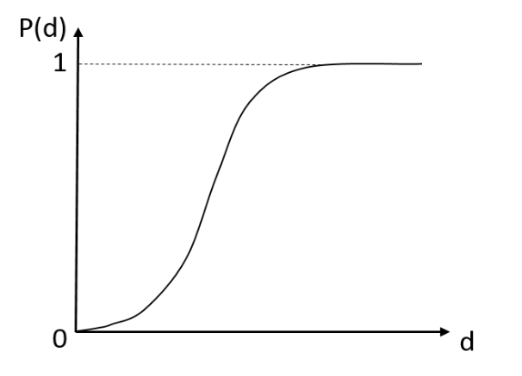


Figure S1: Preference function *P(d)*

1. The sum of the preferences for each object gives a value called the ‘$\pi$ - global preference index’.
2. Outranking flows (ф+) and (ф-) are then computed from the ‘$\pi$ - global preference indices’.
3. A partial rank order (PROMETHEE I) could then be obtained for the observations with three possible outcomes;
4. one observation being preferred over another
5. no difference between the observations or
6. the observations are incomparable
7. A complete rank order (PROMETHEE II) is more often required. This is obtained from the net outranking flow ф (where ф = ф+ – ф-), and it eliminates incomparability.
8. **Qualitative criteria scaling**

Table S2: Molecular weight scale

| **Molecular weight** | **Scale value** |
| --- | --- |
| Very High | 1 |
| High | 2 |
| Medium - High | 3 |
| Medium | 4 |
| Low-Medium | 5 |
| Low | 6 |
| Very Low | 7 |
| No flocculant | 8 |

Table S3: pH scale

| **pH range** | **Scale** | **Scale value** |
| --- | --- | --- |
| 6-8 | Very Good | 1 |
| 8-9 and 5-6 | Good | 2 |
| 9-10 and 4-5 | Poor | 3 |
| <4 and >10 | Very poor | 4 |

1. **Ranking results**

Table S4: Complete PROMETHEE rank order from the 207 observations

| **Observation** | **ф+** | **ф-** | **ф** | **Ranking** |
| --- | --- | --- | --- | --- |
| PF-18 | 0.7885 | 0.0721 | 0.7163 | 1 |
| PF-14 | 0.7588 | 0.071 | 0.6878 | 2 |
| PF-13 | 0.7270 | 0.0775 | 0.6496 | 3 |
| PF-16 | 0.7145 | 0.0865 | 0.6279 | 4 |
| PF-1R1 | 0.6931 | 0.1068 | 0.5863 | 5 |
| DP-15 | 0.7059 | 0.1501 | 0.5558 | 6 |
| PF-17 | 0.6912 | 0.1437 | 0.5475 | 7 |
| DP-13 | 0.6728 | 0.1378 | 0.5350 | 8 |
| PF-20 | 0.6651 | 0.1458 | 0.5192 | 9 |
| DP-11 | 0.6474 | 0.1351 | 0.5123 | 10 |
| DP-6 | 0.6469 | 0.1351 | 0.5118 | 11 |
| PF-19 | 0.6755 | 0.1651 | 0.5104 | 12 |
| PF-9 | 0.6557 | 0.1488 | 0.5069 | 13 |
| PF-1R2 | 0.6354 | 0.1405 | 0.4949 | 14 |
| DP-16 | 0.6461 | 0.1512 | 0.4949 | 15 |
| PF-4 | 0.6376 | 0.1581 | 0.4794 | 16 |
| PF-2 | 0.6298 | 0.1522 | 0.4776 | 17 |
| PF-22 | 0.6239 | 0.1544 | 0.4696 | 18 |
| PF-10 | 0.6370 | 0.1731 | 0.4639 | 19 |
| DP-7 | 0.6210 | 0.1816 | 0.4394 | 20 |
| PF-3 | 0.6485 | 0.2099 | 0.4386 | 21 |
| DP-1 | 0.6151 | 0.1795 | 0.4356 | 22 |
| DP-10 | 0.5996 | 0.1664 | 0.4332 | 23 |
| PF-12 | 0.6223 | 0.1923 | 0.4300 | 24 |
| DP-4 | 0.6341 | 0.2185 | 0.4156 | 25 |
| DP-19 | 0.5833 | 0.2057 | 0.3777 | 26 |
| PF-5 | 0.6402 | 0.2762 | 0.3640 | 27 |
| NF-4 | 0.5935 | 0.239 | 0.3544 | 28 |
| PF-1R4 | 0.5617 | 0.2126 | 0.3491 | 29 |
| DP-17 | 0.5718 | 0.2249 | 0.3470 | 30 |
| PF1-R3 | 0.5596 | 0.2126 | 0.3470 | 31 |
| PF-15 | 0.5825 | 0.2449 | 0.3376 | 32 |
| PF-6 | 0.5534 | 0.2196 | 0.3339 | 33 |
| DP-2 | 0.6122 | 0.2823 | 0.3299 | 34 |
| PG-1 | 0.5697 | 0.2401 | 0.3296 | 35 |
| PF-1 | 0.5532 | 0.2244 | 0.3288 | 36 |
| DP-14 | 0.5617 | 0.2537 | 0.3080 | 37 |
| PF-SS-1 | 0.5473 | 0.2420 | 0.3053 | 38 |
| NF-1 | 0.5732 | 0.2748 | 0.2983 | 39 |
| DP-3R4 | 0.5331 | 0.2356 | 0.2975 | 40 |
| DP-3R5 | 0.5323 | 0.2356 | 0.2967 | 41 |
| DP-3R2 | 0.5318 | 0.2356 | 0.2962 | 42 |
| DP-3R3 | 0.5318 | 0.2356 | 0.2962 | 42 |
| DP-3R6 | 0.5318 | 0.2356 | 0.2962 | 42 |
| NF-3 | 0.5697 | 0.2754 | 0.2943 | 43 |
| DP-3R1 | 0.5299 | 0.2393 | 0.2906 | 44 |
| DP-3 | 0.5291 | 0.2393 | 0.2898 | 45 |
| PG-2 | 0.5494 | 0.2682 | 0.2813 | 46 |
| CP-PF-2 | 0.5502 | 0.2751 | 0.2751 | 47 |
| SF-6 | 0.563 | 0.2922 | 0.2708 | 48 |
| PF-21 | 0.5382 | 0.2703 | 0.2679 | 49 |
| PF-11 | 0.5307 | 0.2735 | 0.2572 | 50 |
| PF-ES-2 | 0.5422 | 0.2869 | 0.2553 | 51 |
| DP-9 | 0.5272 | 0.2823 | 0.2449 | 52 |
| PF-8 | 0.5582 | 0.3141 | 0.2441 | 53 |
| DP-5 | 0.5350 | 0.3085 | 0.2265 | 54 |
| SS-DP-4 | 0.5235 | 0.2978 | 0.2257 | 55 |
| PF-7 | 0.5625 | 0.3462 | 0.2163 | 56 |
| DP-8 | 0.5091 | 0.2938 | 0.2153 | 57 |
| DP-18 | 0.5069 | 0.2933 | 0.2137 | 58 |
| DP-12 | 0.5286 | 0.3192 | 0.2094 | 59 |
| NF-2 | 0.5379 | 0.3363 | 0.2017 | 60 |
| SF-2 | 0.5219 | 0.3237 | 0.1982 | 61 |
| BN-12 | 0.5358 | 0.3456 | 0.1902 | 62 |
| BN-4 | 0.5235 | 0.3774 | 0.1461 | 63 |
| BN-10 | 0.5166 | 0.3814 | 0.1351 | 64 |
| LC-DP-3 | 0.4493 | 0.3365 | 0.1127 | 65 |
| SS-DP-5 | 0.4626 | 0.3558 | 0.1068 | 66 |
| ES-DP-3 | 0.4303 | 0.3339 | 0.0964 | 67 |
| SS-DP-2 | 0.4348 | 0.3584 | 0.0764 | 68 |
| AL-PF-8 | 0.4535 | 0.3793 | 0.0743 | 69 |
| SS-DP-1 | 0.4575 | 0.3908 | 0.0668 | 70 |
| XX-PF-5 | 0.4396 | 0.3790 | 0.0606 | 71 |
| SS-DP-7 | 0.4290 | 0.3718 | 0.0572 | 72 |
| PF-AL-1 | 0.4420 | 0.3868 | 0.0553 | 73 |
| XX-PF-1 | 0.4049 | 0.3571 | 0.0478 | 74 |
| PF-ES-1 | 0.4346 | 0.3876 | 0.0470 | 75 |
| AL-PF-3 | 0.4303 | 0.3862 | 0.0441 | 76 |
| BN-9 | 0.4621 | 0.4268 | 0.0353 | 77 |
| SS-4 | 0.4530 | 0.4193 | 0.0337 | 78 |
| ES-PF-3 | 0.4519 | 0.4191 | 0.0329 | 79 |
| LC-SS-PF-1 | 0.4087 | 0.379 | 0.0296 | 80 |
| SF-3 | 0.4460 | 0.4220 | 0.0240 | 81 |
| XX-PF-1R1 | 0.3985 | 0.3793 | 0.0192 | 82 |
| SF-5 | 0.4463 | 0.430 | 0.0163 | 83 |
| SS-DP-6 | 0.4105 | 0.3980 | 0.0126 | 84 |
| XX-DP-4 | 0.4014 | 0.3985 | 0.0029 | 85 |
| XX-PF-6 | 0.4127 | 0.4180 | -0.0053 | 86 |
| XX-PF-4 | 0.3924 | 0.3977 | -0.0053 | 87 |
| SS-PF-1 | 0.3782 | 0.3894 | -0.0112 | 88 |
| XX-DP-5 | 0.4065 | 0.4207 | -0.0142 | 89 |
| CP-DP-1 | 0.3956 | 0.4108 | -0.0152 | 90 |
| CP-PF-1 | 0.4183 | 0.4348 | -0.0166 | 91 |
| SS-5 | 0.3982 | 0.4172 | -0.019 | 92 |
| ES-DP-2 | 0.3763 | 0.3969 | -0.0206 | 93 |
| SF-1 | 0.4161 | 0.4367 | -0.0206 | 94 |
| BN-13 | 0.4447 | 0.4661 | -0.0214 | 95 |
| XX-PF-7 | 0.3905 | 0.4124 | -0.0219 | 96 |
| CP-PF-6 | 0.4054 | 0.4279 | -0.0224 | 97 |
| XX-DP-1 | 0.3702 | 0.4054 | -0.0353 | 98 |
| BN-8 | 0.4127 | 0.4602 | -0.0475 | 99 |
| SF-7 | 0.4087 | 0.4575 | -0.0489 | 100 |
| XX-PF-2 | 0.3745 | 0.4239 | -0.0494 | 101 |
| ES-5 | 0.3958 | 0.4455 | -0.0497 | 102 |
| CP-PF-3 | 0.3779 | 0.4284 | -0.0505 | 103 |
| AL-PF-2 | 0.3718 | 0.4241 | -0.0524 | 104 |
| ES-DP-4 | 0.3323 | 0.3862 | -0.0540 | 105 |
| XX-DP-6 | 0.3851 | 0.4394 | -0.0542 | 106 |
| CP-DP-5 | 0.3827 | 0.4460 | -0.0633 | 107 |
| BN-11 | 0.3969 | 0.4658 | -0.0689 | 108 |
| ES-DP-1 | 0.3523 | 0.4247 | -0.0724 | 109 |
| AL-PF-6 | 0.3649 | 0.4386 | -0.0737 | 110 |
| XX-DP-7 | 0.3616 | 0.4364 | -0.0748 | 111 |
| SS-2 | 0.3809 | 0.4586 | -0.0777 | 112 |
| AL-PF-11 | 0.3889 | 0.4669 | -0.078 | 113 |
| LC-DP-2 | 0.3785 | 0.4567 | -0.0783 | 114 |
| CP-PF-5 | 0.3702 | 0.4498 | -0.0796 | 115 |
| SF-4 | 0.3846 | 0.4669 | -0.0823 | 116 |
| PH-1 | 0.4057 | 0.4899 | -0.0841 | 117 |
| SS-1 | 0.3977 | 0.4821 | -0.0844 | 118 |
| SS-DP-8 | 0.3868 | 0.472 | -0.0852 | 119 |
| PH-BN-1 | 0.4044 | 0.4917 | -0.0873 | 120 |
| ES-DP-2R1 | 0.3608 | 0.4557 | -0.0948 | 121 |
| AL-DP-1 | 0.3467 | 0.4471 | -0.1004 | 122 |
| ES-DP-4R1 | 0.3496 | 0.4551 | -0.1055 | 123 |
| XX-5 | 0.3755 | 0.4840 | -0.1084 | 124 |
| XX-3 | 0.3803 | 0.4893 | -0.109 | 125 |
| XX-DP-2 | 0.3389 | 0.4543 | -0.1154 | 126 |
| AL-PF-4 | 0.3470 | 0.469 | -0.1221 | 127 |
| CP-DP-2 | 0.3408 | 0.4639 | -0.1231 | 128 |
| XX-PF-3 | 0.3624 | 0.4877 | -0.1253 | 129 |
| AL-DP-8 | 0.3651 | 0.4907 | -0.1255 | 130 |
| SS-DP-3 | 0.3550 | 0.4816 | -0.1266 | 131 |
| LC-DP-4 | 0.3301 | 0.461 | -0.1309 | 132 |
| AL-DP-5 | 0.3333 | 0.4653 | -0.1319 | 133 |
| CP-DP-4 | 0.3419 | 0.4752 | -0.1333 | 134 |
| AL-PF-7 | 0.3515 | 0.4877 | -0.1362 | 135 |
| BN-7 | 0.3632 | 0.4997 | -0.1365 | 136 |
| CP-PF-4 | 0.3424 | 0.4853 | -0.1429 | 137 |
| LC-DP-1 | 0.3178 | 0.4615 | -0.1437 | 138 |
| ES-4 | 0.3237 | 0.4738 | -0.1501 | 139 |
| BN-5 | 0.3606 | 0.5107 | -0.1501 | 140 |
| ES-3 | 0.3104 | 0.4794 | -0.1691 | 141 |
| AL-DP-7 | 0.3245 | 0.4949 | -0.1704 | 142 |
| BN-2 | 0.3405 | 0.5168 | -0.1763 | 143 |
| SF-AL-1 | 0.3344 | 0.5115 | -0.1771 | 144 |
| AL-DP-2 | 0.2981 | 0.4792 | -0.1811 | 145 |
| XX-DP-3 | 0.3328 | 0.5163 | -0.1835 | 146 |
| SS-6 | 0.3443 | 0.5296 | -0.1854 | 147 |
| XX-4 | 0.3218 | 0.508 | -0.1862 | 148 |
| ES-1 | 0.2842 | 0.4792 | -0.195 | 149 |
| AL-DP-6 | 0.3194 | 0.5144 | -0.195 | 150 |
| BN-6 | 0.3387 | 0.5385 | -0.1998 | 151 |
| ES-PF-1 | 0.2901 | 0.4909 | -0.2009 | 152 |
| SS-3 | 0.3168 | 0.5323 | -0.2155 | 153 |
| ES-3R1 | 0.3050 | 0.5243 | -0.2193 | 154 |
| LC-2 | 0.3114 | 0.5342 | -0.2228 | 155 |
| XX-BN-5 | 0.2903 | 0.5144 | -0.2241 | 156 |
| XX-BN-8 | 0.3082 | 0.5331 | -0.2249 | 157 |
| AL-PF-1 | 0.2935 | 0.5208 | -0.2273 | 158 |
| AL-DP-4 | 0.2906 | 0.5230 | -0.2324 | 159 |
| XX-BN-2 | 0.2895 | 0.5248 | -0.2353 | 160 |
| LC-3 | 0.2754 | 0.5166 | -0.2412 | 161 |
| AL-DP-11 | 0.2834 | 0.5283 | -0.2449 | 162 |
| XX-BN-3R1 | 0.2903 | 0.5363 | -0.2460 | 163 |
| XX-BN-3 | 0.2898 | 0.5379 | -0.2481 | 164 |
| SF-AL-3 | 0.3048 | 0.5564 | -0.2516 | 165 |
| XX-BN-1 | 0.2818 | 0.5379 | -0.2561 | 166 |
| ES-2 | 0.2740 | 0.5334 | -0.2593 | 167 |
| AL-8 | 0.2949 | 0.5569 | -0.262 | 168 |
| XX-BN-6 | 0.2967 | 0.5614 | -0.2647 | 169 |
| AL-DP-3 | 0.2647 | 0.5299 | -0.2652 | 170 |
| XX-1 | 0.2804 | 0.5486 | -0.2682 | 171 |
| AL-11 | 0.3064 | 0.5804 | -0.2740 | 172 |
| ES-PF-2 | 0.2743 | 0.5598 | -0.2855 | 173 |
| CP-DP-3 | 0.2639 | 0.5502 | -0.2863 | 174 |
| LC-SS-1 | 0.2593 | 0.5465 | -0.2871 | 175 |
| AL-PF-9 | 0.2938 | 0.5828 | -0.2890 | 176 |
| BN-3 | 0.2906 | 0.5929 | -0.3024 | 177 |
| AL-PF-10 | 0.2452 | 0.5507 | -0.3056 | 178 |
| AL-12 | 0.2476 | 0.5724 | -0.3248 | 179 |
| AL-DP-9 | 0.2687 | 0.5959 | -0.3272 | 180 |
| BN-1 | 0.2660 | 0.6010 | -0.3349 | 181 |
| ES-5R1 | 0.2580 | 0.5935 | -0.3355 | 182 |
| XX-2 | 0.2754 | 0.6132 | -0.3379 | 183 |
| AL-DP-10 | 0.219 | 0.5716 | -0.3526 | 184 |
| XX-BN-4 | 0.2553 | 0.6119 | -0.3566 | 185 |
| AL-7 | 0.2575 | 0.6178 | -0.3603 | 186 |
| LC-1 | 0.2244 | 0.5871 | -0.3627 | 187 |
| LC-4 | 0.2366 | 0.6058 | -0.3691 | 188 |
| SF-AL-6 | 0.2623 | 0.633 | -0.3707 | 189 |
| SF-AL-2 | 0.2361 | 0.6082 | -0.3721 | 190 |
| AL-14 | 0.2334 | 0.6114 | -0.3779 | 191 |
| SF-AL-5 | 0.2385 | 0.6311 | -0.3926 | 192 |
| AL-17 | 0.2441 | 0.6370 | -0.3929 | 193 |
| AL-18 | 0.2252 | 0.6295 | -0.4044 | 194 |
| AL-1 | 0.2174 | 0.6311 | -0.4137 | 195 |
| XX-BN-7 | 0.2075 | 0.6343 | -0.4268 | 196 |
| AL-4 | 0.2401 | 0.6699 | -0.4298 | 197 |
| AL-PF-5 | 0.2078 | 0.6458 | -0.4380 | 198 |
| AL-6 | 0.2097 | 0.6504 | -0.4407 | 199 |
| SF-AL-4 | 0.2257 | 0.6720 | -0.4463 | 200 |
| AL-2 | 0.2374 | 0.6886 | -0.4511 | 201 |
| AL-3 | 0.203 | 0.6928 | -0.4899 | 202 |
| AL-5 | 0.1723 | 0.6952 | -0.523 | 203 |
| AL-10 | 0.1731 | 0.7105 | -0.5374 | 204 |
| AL-13 | 0.1757 | 0.7206 | -0.5449 | 205 |
| AL-9 | 0.1568 | 0.7535 | -0.5967 | 206 |
| AL-15 | 0.0705 | 0.7989 | -0.7284 | 207 |

Average permeate flux: Permeate flux describes the quantity of permeate produced during microalgae separation per unit of time and screen area. The permeate flux can be obtained using the equation below;

$$J=\frac{Q_{P}}{A_{system}}$$

where *J* is the permeate flux (L/m^2^.h), *Q_P_* is the permeate flow (L/h) and *A_system_* is the surface area of the filter screen.

For the BDDJ optimum conditions the permeate flux was determined as below, with *Q_P_* = 12 L/h and *A_system_* = 7.86 x 10^-3^.

$$J=\frac{12}{{7.8x10}^{-3}}$$

$J=1527 L/m^{2}.h$

1. **References**

1. Keller HR, Massart DL, Brans JP. Multicriteria decision making: A case study. Chemom Intell Lab Syst. 1991;11:175–89. doi:10.1016/0169-7439(91)80064-W.

2. Kokot S, Ayoko GA. CHEMOMETRICS AND STATISTICS | Multicriteria Decision Making. In: Encyclopedia of Analytical Science. Elsevier; 2005. p. 40–5. doi:10.1016/b0-12-369397-7/00747-0.

3. Kokot S, Grigg M, Panayiotou H, Phuong TD. Data Interpretation by some Common Chemometrics Methods. Electroanalysis. 1998;10:1081–8. doi:10.1002/(SICI)1521-4109(199811)10:16<1081::AID-ELAN1081>3.0.CO;2-X.

4. Settle S, Goonetilleke A, Ayoko GA. Determination of surrogate indicators for phosphorus and solids in urban stormwater: Application of multivariate data analysis techniques. Water Air Soil Pollut. 2007;182:149–61.

5. Ayoko GA, Singh K, Balerea S, Kokot S. Exploratory multivariate modeling and prediction of the physico-chemical properties of surface water and groundwater. J Hydrol. 2007;336:115–24. doi:10.1016/j.jhydrol.2006.12.013.

6. Brans JP, De Smet Y. PROMETHEE methods. Int Ser Oper Res Manag Sci. 2016;233:187–219.
